# Genome wide association study reveals novel associations with face morphology

**DOI:** 10.1371/journal.pone.0299660

**Published:** 2025-02-10

**Authors:** Aamer Alshehhi, Mira Mousa, Guan K. Tay, Naoufel Werghi, Habiba AlSafar

**Affiliations:** 1 Department of Biomedical Engineering, Khalifa University of Science and Technology, Abu Dhabi, United Arab Emirates; 2 Center for Biotechnology, Khalifa University of Science and Technology, Abu Dhabi, United Arab Emirates; 3 Division of Psychiatry, Medical School, the University of Western Australia, Crawley, Western Australia, Australia; 4 School of Medical and Health Sciences, Edith Cowan University, Joondalup, Western Australia, Australia; 5 Department Electrical Engineering and Computer Science, C2PS, Khalifa University of Science and Technology, Abu Dhabi, United Arab Emirates; 6 Emirates Bio-Research Center, Ministry of Interior, Abu Dhabi, United Arab Emirates; Government College University Faisalabad, PAKISTAN

## Abstract

Genome-wide association studies (GWAS) on the Middle Eastern population, including the United Arab Emirates (UAE), have been relatively limited. The present study aims to investigate genotype-face morphology associations in the UAE population through Genome Wide Association Studies (GWAS). Phenotypic data (44 face measurements) from 172 Emiratis was obtained through three-dimensional (3D) scanning technology and an automatic face landmarking technique. GWAS analysis revealed associations of 19 genetic loci with six face features, 14 of which are novel. The GWAS analysis revealed 11 significant relationships between 44 face parameters and 242 SNPs, exceeding the GWAS significance threshold. These phenotypes were previously associated with body height, craniofacial defects, and facial characters. The most significant associations of these genetic variations were related to six main facial features which were facial convexity, left orbital protrusion, mandibular contour, nasolabial angle D, inferior facial angle B, and inferior facial angle A. To the best of our knowledge, this is the first GWAS study to investigate the association of SNP variations with face morphology in the Middle Eastern population.

## Introduction

Face morphology undergoes complex processes during embryonic development and is subject to genetic variations and environmental factors. Craniofacial anomalies are one of the most common birth defects, including down syndrome, Treacher Collins syndrome, cleft palate and cleft lip, and hemifacial microsomia [1]. Understanding the effect of genes on facial morphology, through the use of three-dimensional (3D) imaging technologies, is essential in advancing clinical craniofacial studies through early diagnosis of possible syndromes, pharmacogenomics and advancing medical treatments.

The heritability of craniofacial morphology is 0.80 for parents and offspring, and 0.41–0.86 based on twin studies, suggesting that face morphology is strongly determined by genetic factors [2]. Initial studies to identify genetic markers affecting face morphology were conducted on patients diagnosed with genetic syndromes. Several craniofacial genes that affect face morphology have been identified, such as: *FOXE1* in locus 9q22 is associated with the risk of orofacial clefts, and *GREM1* in locus 15q13 is associated with cleft lip and palate [3]. Genome Wide Association Studies (GWAS) on European individuals identified five genes that affect face features: *TP63* gene in locus 3q28 showed association with the distance between eyeballs, *C5orf5N* gene in locus 5q35.1 is associated with nasion position, *PRDM16* located in loci 1p36.23-p36.33 are associated with nose width and nose height, *COL17A1* in locus 10q24.3 is associated with the distance between eyeballs and nasion, and *PAX3* located in 2q35 is associated with the distance between the eyeballs and nasion [4, 5]. Another study conducted on Americans from European ancestry confirmed the effect of an additional six genes involved in craniofacial development on facial features, such as: *PAX1* in locus 20p11.22, *PAX9* in locus 14q21.1, *ALX3* in locus Xq13.2, *HDAC8* in the X-exome, *MAFB* in locus 20q12, *MIPOL1* in locus 14q21.1 [6]. In the Latin-American population, *DCHS2*, *RUNX2*, *GLI3*, *PAX1* and *EDAR* genes have been associated to nose and chin related traits [7, 8]. Skeletal analysis has indicated that the overall variation between population groups is 10%, underscoring the need for expanded genetic studies on facial morphology [7]. Given that variations in facial morphology exist among populations, further research is crucial to understand the genetic underpinnings of these variations.

Exploring the genetic associations with face features in diverse population groups may lead to the discovery of novel genetic markers. In this study, we conducted a GWAS analysis on a cohort of 172 subjects from the United Arab Emirates (UAE) to identify genetic markers that are associated with 44 face measurements (15 eye related measurements, 13 nose related measurements, eight mouth related measurements, and six chin measurement). To the best of our knowledge, this is the first GWAS study that investigates the association of genotype-face morphology in a Middle Eastern population.

## Results

A total of 172 Emirati participants (89 males and 83 females) were recruited for this study. The subjects’ age range was 18–49 years old, with an average age of 22.5 ± 4.8. Demographic information of the participants was collected through a questionnaire, which included information regarding age, birthplace, area of settlement within UAE, ethnic background, and general health questions. Measurements of height (165.83 cm ± 9.21), weight (71.47 kg ± 18.29), and body mass index (BMI) (25.83 ± 5.44) were also obtained from the participants. A total of 44 linear and angular face measurements were obtained from 3D face scans of the participants. S1 Table in S1 File demonstrates the descriptive statistics of 44 linear and angular face measurements of all subjects.

In our study, linear regression model was used to test for the association between 325,984 SNPs, 159 participants and 44 face measurements. To avoid confounding effects from demographic factors, the association analysis was adjusted for covariates such as: age, gender, BMI, and population stratification. Manhattan plots for the significant GWAS associations and Quantile-Quantile (QQ) plots are shown in S1-S88 Figs in S1 File. An admixture informed PCA was plotted for the 159 participants from this study, represented as fuchsia in the PCA plot, merged with 1,115 participants from the HapMap 3 project. As demonstrated in S89 Fig in S1 File, the participants were clustered together to avoid technical confounders and population stratification.

**Fig 1:**
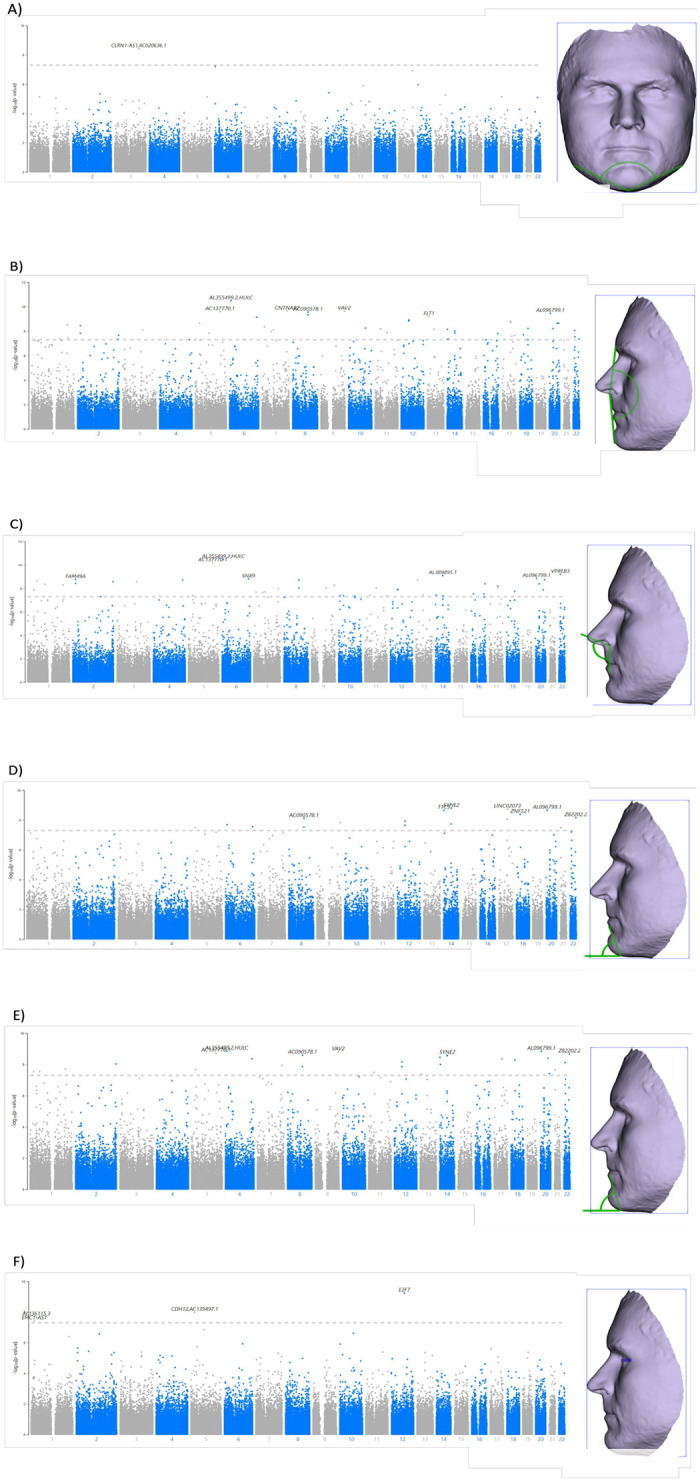
Manhattan plot shows the associations between the (-log10[*P*]) on the (y-axis) and the genotyped SNPs from the study (each dot represent a SNP according to the chromosomal position (numbered from 1–22) on the (x-axis). The horizontal dashed grey line shows the genome-wide significance threshold which is (*p* < 5×10^−8^). Despite this threshold, the implemented Bonferroni correction in this study was (*p* < 1.5×10^−7^), which is represented in a blue line. On the right side of each Manhattan plot, it includes the highlighted measurement of the respective facial trail, which was obtained from the subject reference list (Rich) from Cliniface software. The following facial traits are demonstrated in the figure: A) Mandibular contour; B) Facial convexity (Angle); C) Nasolabial angle D; D) Inferior facial angle A; E) Inferior facial angle B and GWAS association results; and F) Left orbital protrusion (Angle).

Of the 44 facial traits, 11 traits exhibited 242 SNPs that surpassed the significance threshold proposed by the GWAS at a statistically significant level (1.5×10^−7^), including phithral width (n = 1 SNP), Mandibular Contour (n = 3 SNPs), Facial Convexity (n = 80 SNPs), upper lip circularity (n = 1 SNP), outer canthal nasal angle (n = 2 SNPs), Left Nasal Ala Length (n = 1 SNP), Nasofrontal Angle (n = 1 SNP), nasolabial angle D (n = 73 SNPs), inferior facial angle A (n = 30 SNPs), inferior facial angle B (n = 45 SNPs), and left orbital protrusion (n = 5 SNPs). Of those, six traits, mandibular contour (Fig 1A), facial convexity (Fig 1B), nasolabial angle D (Fig 1C), inferior facial angle A (Fig 1D), inferior facial angle B (Fig 1E) and left orbital protrusion (Fig 1F), have SNPs with previous associations to face morphology [16–24]. These morphologies include associations to body height, craniofacial defects (microcephaly), and multiple facial features, such as, ear morphology (antitragus size, ear lobe attachment, and folding of antihelix), vertical position of orbits relative to midface, cleft lip and palate (orofacial cleft), facial morphology (lower face morphology measurements, breadth of lateral portion of upper face, projection of the nose and width of nasal floor, nasal bridge angle, philtrum width, right exocanthion-left cheilion distance, and facial dysmorphism).

For body height, a total of 17 genetic locus across 16 genes were identified in the following phenotypes: facial convexity, left orbital protrusion, mandibular contour, nasolabial angle D, inferior facial angle B, and inferior facial angle A (Table 1) [9–12].

**Table 1:** Genetic markers surpassing GWAS significance threshold in association with face morphology and phenotype. Each marker is depicted alongside its minor allele and frequency, beta values, and p-values, providing a detailed overview of their genetic contributions to facial features.

Gene	Cytogenic Region	SNP	Minor Allele	MAF	Phenotype	Beta	P-Value
**Body Height**							
***ADGRL2***	1p31.1	rs4477328	A	0.07	Nasolabial Angle D	-21.42	1.07 x 10^−7^
***LRRC52-AS1***	1q23.3-q24.1	rs10494444	A	0.06	Facial Convexity	-33.46	4.16 x 10^−8^
***HDAC4***	2q37.3	rs61333513	A	0.06	Facial Convexity	-33.6	2.15 x 10^−8^
					Nasolabial Angle D	-26.82	2.69 x 10^−9^
					Inferior Facial Angle A	-14.67	8.90 x 10^−8^
					Inferior Facial Angle B	-14.52	9.26 x 10^−9^
***GRID2***	4q22.1-q22.2	rs62307849	A	0.08	Inferior Facial Angle B	-11.93	1.09 x 10^−7^
***CDH12***	5p14.3	rs3020548	C	0.08	Left Orbital Protrusion	-3.56	1.03 x 10^−8^
***ARSB***	5q14.1	rs34902218	A	0.08	Facial Convexity	-28.28	1.34 x 10^−7^
***F13A1***	6p25.1	rs9379015	A	0.07	Mandibular Contour	71.36	6.10 x 10^−8^
***EGFR***	7p11.2	rs17172428	G	0.05	Facial Convexity	-35.92	1.10 x 10^−8^
					Nasolabial Angle D	-26.64	2.22 x 10^−8^
***LCOR***	10q24.1	rs72819856	A	0.07	Facial Convexity	-32.51	5.55 x 10^−9^
					Nasolabial Angle D	-23.01	6.00 x 10^−8^
					Inferior Facial Angle B	-12.87	5.92 x 10^−8^
***PTPRB***	12q15	rs10879160	G	0.06	Facial Convexity	-32.6	6.65 x 10^−8^
		rs10879160	G	0.06	Inferior Facial Angle B	-13.67	8.57 x 10^−8^
***E2F7***	12q21.2	rs11116823	G	0.05	Left Orbital Protrusion	-4.413	5.74 x 10^−10^
***FLT1***	13q12.3	rs78305106	G	0.05	Facial Convexity	-39.83	6.32 x 10^−10^
					Nasolabial Angle D	-29.38	1.82 x 10^−9^
					Inferior Facial Angle B	-15.13	4.05 x 10^−8^
***SYNE2***	14q23.2	rs72718380	G	0.05	Facial Convexity	-36.62	1.37 x 10^−8^
					Nasolabial Angle D	-26.79	4.37 x 10^−8^
					Inferior Facial Angle A	-17.6	1.57 x 10^−9^
					Inferior Facial Angle B	-16.14	2.66 x 10^−9^
***SAMD14***	17q21.33	rs73330581	A	0.06	Facial Convexity	-36.35	1.53 x 10^−9^
					Nasolabial Angle D	-26.59	5.78 x 10^−9^
					Inferior Facial Angle A	-15.84	8.71 x 10^−9^
					Inferior Facial Angle B	-14.95	4.54 x 10^−9^
		rs9915064	A	0.06	Facial Convexity	-36.35	1.53 x 10^−9^
					Nasolabial Angle D	-26.59	5.78 x 10^−9^
					Inferior Facial Angle A	-15.84	8.71 x 10^−9^
					Inferior Facial Angle B	-14.95	4.54 x 10^−9^
***LINC02073***	17q21.33	rs28716452	A	0.06	Facial Convexity	-36.52	1.97 x 10^−9^
					Nasolabial Angle D	-26.63	8.36 x 10^−9^
					Inferior Facial Angle A	-16.65	1.81 x 10^−9^
					Inferior Facial Angle B	-15.14	4.22 x 10^−9^
***ZNF521***	18q11.2	rs45475191	G	0.07	Facial Convexity	-33.29	1.93 x 10^−8^
					Nasolabial Angle D	-23.9	1.11 x 10^−7^
					Inferior Facial Angle A	-15.78	4.07 x 10^−9^
					Inferior Facial Angle B	-14.57	5.11 x 10^−9^
**Facial Morphology**							
***LINC01780***	1p12	1:119872141	G	0.05	facial convexity	-31.4	1.24 x 10^−7^
					nasolabial angle D	-23.94	9.39 x 10^−8^
***FYB2***	1p32.2	rs4912226	G	0.06	facial convexity	-32.66	8.72 x 10^−8^
					nasolabial angle D	-27.3	2.17 x 10^−9^
					inferior facial angle B	-14.26	2.89 x 10^−8^
***HHAT***	1q32.2	rs12029929	G	0.05	facial convexity	-37.19	3.03 x 10^−9^
					nasolabial angle D	-27.76	5.06 x 10^−9^
					inferior facial angle B	-14.96	1.98 x 10^−8^
***CSRNP3***	2q24.3	rs73029902	A	0.07	facial convexity	-30.81	1.10 x 10^−7^
					nasolabial angle D	-23.83	5.16 x 10^−8^
***RASGRF2***	5q14.1	rs26903	A	0.24	Left Orbital Protrusion	-2.09	1.44 x 10^−7^
***CNTNAP2***	7q35-q36.1	rs75898451	A	0.05	facial convexity	-40.39	2.24 x 10^−10^
					nasolabial angle D	-27.88	1.04 x 10^−8^
					inferior facial angle A	-15.86	7.51 x 10^−8^
					inferior facial angle B	-15.55	1.12 x 10^−8^
		rs7810054	G	0.07	nasolabial angle D	-23.13	1.37 x 10^−7^
***CSMD1***	8p23.2	rs12114533	G	0.06	facial convexity	-31.68	8.09 x 10^−8^
					nasolabial angle D	-24.19	5.78 x 10^−8^
***HS3ST4***	16p12.1	rs17774701	G	0.07	facial convexity	-30.62	1.14 x 10^−7^
***ZFHX3***	16q22.2-q22.3	rs55989215	C	0.07	inferior facial angle A	-13.99	1.00 x 10^−7^
					inferior facial angle B	-12.9	1.27 x 10^−7^
***MYO18B***	22q12.1	rs34427191	A	0.05	facial convexity	-33.06	2.81 x 10^−8^
					inferior facial angle B	-13.51	8.67 x 10^−8^

Gene *LINC01780* in 1p12 was associated to facial convexity (p = 1.24 x 10^−7^) and nasolabial angle D (p = 9.39x10^-8^), and has been previously associated to multiple facial features including lower face morphology measurement and ear morphology, such as antitragus size, ear lobe attachment, and folding of antihelix. SNP rs34427191 in gene *MYO18B* was associated to facial convexity (p = 2.81x10^-8^) and inferior facial angle B (p = 8.67x10^-8^), which have been previously associated to facial morphology, specifically the vertical position of orbits relative to midface and facial dysmorphism [13–16]. Gene *RASGRF2* in SNP rs26903 in left orbital protrusion (p = 1.44x10^-7^) has been associated with cleft lip and palate, and facial morphology, such as breadth of lateral portion of upper face [9, 16, 17]. In gene *ZFHX3*, rs55989215 in inferior facial angle A (p = 1.00 x 10^−7^) and inferior facial angle B (p = 1.27x 10^−7^) has been associated to orofacial cleft and facial morphology dysmorphia [16, 18].

Gene *CNTNAP2* has been linked to facial morphology, specifically the projection of the nose and width of the nasal floor [16]. The SNP rs75898451 within this gene has been demonstrated associations with facial convexity (*p* = 2.24 x 10^−10^), nasolabial angle D (*p* = 1.04x10^-8^), inferior facial angle A (*p* = 7.51x10^-8^), and inferior facial angle B (*p* = 1.12x10^-8^), and SNP rs7810054 is associated to nasolabial angle D (*p* = 1.37x10^-7^). Gene *CSMD1* with SNP rs12114533 associated to facial convexity (p = 8.09x10^-8^) and nasolabial angle D (p = 5.78x10^-8^) has been associated to facial morphology, specifically the right exocanthion-left cheilion distance [19]. In *HHAT* gene, SNP rs12029929 associated to facial convexity (p = 3.03x10^-9^), nasolabial angle D (*p* = 5.06x10^-9^), and inferior facial angle B (*p* = 1.98x10^-8^) is associated to craniofacial defects, cleft lip pathogenesis, microcephaly, and embryogenesis of palate development [20–23]. SNP rs17774701 located in gene *HS3ST4* linked to facial convexity (*p* = 1.14x10^-7^), is associated to nose morphology, specifically the nasal bridge angle [19].

The *CSRNP3* gene in SNP rs73029902 associated to facial convexity (*p* = 1.10x10^-7^) and nasolabial angle D (*p* = 5.16x10^-8^) has been associated to facial morphology, specifically at the philtrum width, bone mineral density, and height [9, 16, 24, 25]. SNP rs4912226 in gene *FYB2* in facial convexity (*p* = 8.72x10^-8^), nasolabial angle D (*p* = 2.17x10^-9^), and inferior facial angle B (*p* = 2.89x10^-8^) were associated to unilateral cleft lip [26].

## Discussion

During the early stages of development, craniofacial morphogenesis and facial patterns are established through the vital functions of cranial neural crest cells, genetic variants, transcriptional regulatory networks and epigenetic landscapes [32]. In our study on the population of the UAE, we examined the genetic links to particular facial measures. We identified that 11 out of 44 face characteristics demonstrated 242 SNPs that were statistically significant, above the GWAS suggested significance threshold.

Furthermore, our research supports previous investigations, as demonstrated by six facial traits —philtral width, mandibular contour, facial convexity, nasolabial angle D, inferior facial angle A, inferior facial angle B, and left orbital protrusion—surpassing the GWAS significance threshold in their association with face morphology. Facial convexity, which is determined by the protrusion of the eye and the shape of the lower jaw, is measured by the subnasal angle formed by the side of the forehead and is affected by the position of the chin (pogonion) and the area between the eyebrows (glabella). The mandibular contour, which defines the form of the lower face, is connected to the facial convexity. This connection influences the angles of the lower face, and the overall structure of the face. Additionally, the orbital protrusion impacts the depth and shape of the face, which in turn has an impact on the facial convexity [27]. Finally, the nasolabial angle, which represents the aesthetics of the middle part of the face, is affected by the shape of the lower jaw and, in turn, impacts the overall curvature of the face [28]. These factors among others contribute to the complex structure of the face [29].

Various facial dimensions, namely the angles of the face, were shown to be associated with hereditary variables that determine body height [30–34]. Several investigations have demonstrated that the proportions of body height are intricate and influenced by multiple genes [35, 36]. The study conducted by Chan et al. (2015) offers a detailed comprehension of the role played by polygenic variables in shaping body proportions. They demonstrated that genetic variations that affect the overall height, can also have diverse effects on other body parts, such as the face [29].

Multiple studies have examined the genetic basis of face structure and how it relates to the distribution of body fat [37]. The *F13A1* gene was shown to be significantly correlated with Mandibular Contour in our study, indicating that its biological relevance in adipocytes goes beyond what has been previously known. This is consistent with research that links *F13A1* to changes in body weight and fat mass. The link between *F13A1* and mandibular anatomy suggests an intricate relationship between genetic variables that influence face development and metabolic traits [38].

Additionally, the *ARSB* gene’s association with face convexity, as suggested by SNP rs34902218, was previously linked to eyelid sagging [39]. In this study, we have shown connections between the *EGFR* gene and face dimensions, specifically with the SNP rs17172428. This finding supports the importance of the *EGFR* gene in early craniofacial development and palate closure [40]. In our investigation, we found that the *CDH12* gene (SNP rs3020548) was linked to orbital protrusion. Previous research has also shown that this gene is involved with Craniofacial-Deafness-Hand Syndrome [41]. Moreover, SNP rs11116823, located in the *E2F7* gene, has been linked to left orbital protrusion and craniofacial abnormalities [42].

*HHAT* gene was associated with facial convexity, as well as significant roles in the development of the embryo, body size, and neurological system. Remarkably, facial convexity was associated to another gene, *EGFR*. This protein-coding gene has a vital function in the growth of different organs during both the embryonic and postnatal periods [36]. The gene *DBX2* is linked to facial convexity and skeletal phenotype [43, 44]. Additionally, the facial convexity was found to be linked to the *SAMD14* gene, which has previously been identified as being connected with central nervous system lymphoma and hematologic malignancy [45, 46]. These observations collectively emphasize the complex genetic coordination involved in face development.

In addition, our work emphasizes the wide array of genetic factors that affect face structure, as demonstrated by the connections between numerous facial characteristics and genes such as *F13A1*, *FLT1*, *GRID2*, and *HDAC4*. These findings are consistent with previous studies, emphasizing the complex role of genetic variables in the formation of face features [47]. For instance, the genetic correlations of disorders such as cleft lip, craniofacial abnormalities, and microcephaly, specifically related to genes like *ZFHX3* and *HHAT*, are consistent with previous research findings [18].

Our study recognizes possible limitations. From a methodological perspective, the sample size did not meet the expected power analysis to identify genome-wide significant variations. Notwithstanding this constraint, we applied the Bonferroni correction method to detect genetic variations that were shown to have statistical significance at a genome-wide level. In order to account for any correlations across characteristics, we applied the eigenvalue-greater-than-one method [48, 49]. Furthermore, it is crucial to conduct future research on a larger cohort that specifically target individuals of Middle Eastern ancestry in order to ensure the validity and reliability of the findings. Performing pathway analysis on different tissues associated with the same genes, and functional validation, may elucidate the correlation between these genes and characteristics related to body height as well as the distribution of body fat.

## Conclusion

The GWAS study of 44 facial traits identified 11 traits with 242 statistically-significant SNP, in which six facial traits —philtral width, mandibular contour, facial convexity, nasolabial angle D, inferior facial angle A, inferior facial angle B, and left orbital protrusion—included SNPs that were previously associated to face morphology in other populations. This study contributes to the existing body of information on craniofacial genetics, particularly in relation to the population of the United Arab Emirates. It strengthens the data obtained from worldwide GWAS research findings. The differences found amongst communities may be attributed to their unique genetic compositions, highlighting the need for further investigation into the population-specific genetic factors that determine face morphology.

## Material and methods

### Sample collection

Prior to enrollment, participants voluntarily provided their written informed consent, which had been approved by the Institutional Review Board (IRB) of Khalifa University (Reference: H18-024). The study participants were recruited from Khalifa University Campus in the Emirate of Abu Dhabi. The inclusion criteria were participants between the ages of 18–49, Emirati descent, and able to provide an informed consent and complete the survey and face measurement profile. This cross-sectional study recruited a total of 172 participants.

The Oragene OFR-500 kit was utilized for saliva sample collection from all participants. DNA extraction was performed according to the recommended protocol (DNA Genotek, Ottawa, Canada). DNA quantification was performed using DS 11 FX Fluorometer dsDNA Broad Range Assay (Denovix Inc., Wilmington, DE, United States) [29]. Nanodrop 2000C Spectrometer was used to confirm the quality of the extracted DNA (ThermoFisher, Wilmington, USA).

### Facial imaging and landmarking

Face scanning was performed using the Artec Eva Scanner, which scans the soft tissue of the face using a structured light 3D scanning technology [30]. All participants maintained neutral facial expression during face scans. For the purpose of this research, the investigated face area includes the frontal view (forehead hairline to chin) and lateral view (ear to ear). Default scanning parameters were applied, except for the sensitivity, which was raised to the maximum level to obtain images of the highest resolution (0.5mm) / 3D point accuracy (0.1mm—0.03% over 100cm). ArtecStudio software was then used to process all images using default parameters, as the Autopilot mode generates 3D meshes of high resolution by applying post-processing algorithms [31].

Cliniface open access software (version 6.0.4.210510) (Perth, Western Australia) was used for face automatic landmarking and face measurement extraction. The software applies Meshmonk algorithm for automatic facial landmark detection by non-rigidly transforming an anthropometric mask (AM) to the subject’s face [14]. A total of 69 landmarks were automatically positioned on the face; 25 two-sided landmarks and 19 unilateral landmarks, which were also confirmed manually by the analyst according to the reference guiding tool of the software. Using the measurements browser tool in the software, a total of 44 linear and angular face measurements were extracted that were investigated in this study (S1 Table in S1 File).

### Genotyping and quality control

Genotyping of the participants’ DNA for GWAS was utilized using microarray technology with Infinium Global Screening Array-24 v3.0 BeadChip, which has 654,027 SNPs [13, 32]. Illumina’s iScan System was used for the chips’ scanning process [33]. All hybridization and scanning procedures were undertaken following the manufacturer’s recommended protocol.

The primary genotyping analysis was run on the GenomeStudio software version (2.02.5.0) [34]. The recommended amount of 200 ng of genomic DNA was used in the genotyping process. The genotyped samples were mapped against the GRCh38 Genome Reference Consortium Human Build 38 with call rate > 99%. SNPs quality control (QC) threshold using Plink 1.9 was set as the following: minor allele frequency (MAF) > 0.05, genotype missingness per marker > 0.1, missingness per individual > 0.1, and Hardy Weinberg Equilibrium (HWE) > 0.0001. Sex information accuracy was genetically confirmed for all samples. Elevated missing data rates (outlying heterozygosity rate) was confirmed to be 100%. Non-autosomal markers were removed from the analysis. Initially, 172 Emiratis were recruited in the study, but four of them were excluded due to high relatedness and inbreeding coefficient (PI_HAT > 0.5), and nine were excluded as outliers in the principal component analysis (PCA) ancestry. Finally, 159 participants and 325,984 variants passed QC and were included for the analysis of this study.

### Statistical analysis

To determine the association between SNPs and the 44 linear and angular face measurements, multiple linear regressions using the additive model were performed with/without adjustment for age, sex, BMI, and the first two principal components (PCs) for population stratification. LocusZoom.js 0.12.0 plotting tool was used to visualize the GWAS results using Manhattan and Q-Q plots. For any associations made, the GC lambda (λ) of 0.5 = (1± 0.05) was conducted.

Genetic variants were reported to have genome-wide statistical significance with a threshold of *p* < 1.5×10^−7^ (i.e., Bonferroni correction for *p* < 0.05 divided by 325,984 SNPs). Given that most of the traits may be correlated, we utilized the eigenvalue-greater-than-one rule by Li and Ji, with an effective number of independent traits was 44, and a study-wise statistical significance at *p* < 3.41×10^−9^ (*p* < 1.5×10^−7^ divided by 44 independent tests) [48, 49].

## Supporting information

S1 File (DOCX)
