# Supplementary material for: Genome wide association study reveals novel associations with face morphology
Source: PLoS One. 2025 Feb 10;20(2):e0299660. doi: 10.1371/journal.pone.0299660 (PMC11809905; doi:10.1371/journal.pone.0299660)
Supplement: S1 File — (DOCX) [file pone.0299660.s001.docx]

Supplementary File

**Supplementary Table 1.** Descriptive statistics of 44 linear and angular face measurements of all subjects. SD: Standard deviation.

| **Phenotype** | **Mean** | **SD** | **Median** | **Minimum** | **Maximum** |
| --- | --- | --- | --- | --- | --- |
| **Outercanthal Width** | 95.62 | 4.63 | 94.95 | 86.60 | 108.72 |
| **Innercanthal Width** | 35.56 | 2.58 | 35.38 | 29.12 | 42.65 |
| **Left Palpebral Fissure Length** | 31.75 | 2.40 | 31.42 | 27.28 | 39.07 |
| **Right Palpebral Fissure Length** | 31.50 | 2.42 | 31.38 | 21.09 | 38.97 |
| **Facial Height** | 122.89 | 8.93 | 122.69 | 104.88 | 153.58 |
| **Bizygomatic Width** | 142.44 | 8.16 | 141.85 | 118.74 | 161.23 |
| **Mandibular Width** | 112.06 | 9.09 | 110.57 | 91.18 | 141.69 |
| **Bifrontal Width** | 123.93 | 6.86 | 123.42 | 109.23 | 143.43 |
| **Lower Vermilion Height** | 9.62 | 1.40 | 9.72 | 3.96 | 13.37 |
| **Philtral Length** | 13.11 | 1.52 | 13.13 | 7.68 | 16.71 |
| **Labial Fissure Width** | 51.25 | 3.37 | 51.06 | 43.69 | 59.85 |
| **Philtral Width** | 13.72 | 1.17 | 13.47 | 11.66 | 19.74 |
| **Upper Lip Height** | 20.35 | 2.01 | 20.51 | 13.14 | 25.72 |
| **Upper Vermilion Height** | 8.13 | 1.18 | 8.12 | 4.77 | 11.32 |
| **Subnasal Width** | 16.07 | 1.76 | 15.93 | 10.94 | 24.87 |
| **Nasal Width** | 33.53 | 2.79 | 33.05 | 25.28 | 41.16 |
| **Columella Length** | 9.31 | 1.14 | 9.30 | 5.82 | 13.98 |
| **Nasal Bridge Length** | 45.95 | 2.61 | 46.04 | 39.29 | 52.77 |
| **Nasal Height** | 52.18 | 3.01 | 51.88 | 45.63 | 60.16 |
| **Nasal Root Width** | 12.91 | 1.72 | 12.57 | 9.58 | 18.89 |
| **Interpupillary Distance** | 64.68 | 3.34 | 64.53 | 57.68 | 77.09 |
| **Left Palpebral Fissure Inclination** | 1.03 | 3.33 | 1.21 | -8.64 | 11.44 |
| **Right Palpebral Fissure Inclination** | 1.78 | 3.74 | 1.72 | -10.91 | 16.02 |
| **Left Palpebral Fissure Height** | 12.21 | 1.59 | 12.09 | 7.89 | 16.07 |
| **Right Palpebral Fissure Height** | 12.23 | 1.64 | 12.22 | 7.79 | 16.50 |
| **Mandibular Contour** | -93.36 | 68.62 | -114.34 | -136.43 | 160.44 |
| **Facial Convexity** | 159.09 | 27.51 | 160.96 | -179.23 | 175.40 |
| **Nasal Protrusion** | 19.00 | 1.54 | 18.96 | 13.17 | 23.99 |
| **Upper Lip Circularity** | 45.84 | 6.23 | 44.71 | 34.13 | 69.65 |
| **Outer Canthal, Nasal Angle** | -81.07 | 55.62 | -97.39 | -143.45 | 106.51 |
| **Cutaneous Lower Lip Height** | 9.76 | 2.27 | 9.24 | 6.21 | 17.27 |
| **Left Nasal Ala Length** | 32.42 | 2.19 | 32.28 | 26.86 | 39.00 |
| **Right Nasal Ala Length** | 32.30 | 2.31 | 32.11 | 26.63 | 38.40 |
| **Nasofrontal Angle** | 146.86 | 7.49 | 147.06 | 130.39 | 163.95 |
| **Nasal Angle** | 117.54 | 4.99 | 117.83 | 100.67 | 130.47 |
| **Nasolabial Angle A** | 105.74 | 14.02 | 106.12 | 75.80 | 167.31 |
| **Total Facial Convexity** | 136.77 | 4.96 | 136.61 | 122.81 | 152.45 |
| **Nasolabial Angle D** | 116.66 | 20.78 | 117.77 | -137.47 | 139.76 |
| **Inferior Facial Angle A** | 61.49 | 12.77 | 62.47 | -77.89 | 79.43 |
| **Inferior Facial Angle B** | 66.70 | 11.79 | 67.51 | -70.37 | 78.86 |
| **Inferior Facial Angle C** | 42.91 | 6.57 | 42.98 | 17.33 | 75.42 |
| **Inferior Facial Angle D** | 51.71 | 5.48 | 51.78 | 32.56 | 84.91 |
| **Left Orbital Protrusion** | 19.67 | 3.32 | 19.88 | 0.00 | 26.79 |
| **Right Orbital Protrusion** | 20.38 | 2.29 | 20.42 | 8.65 | 26.33 |


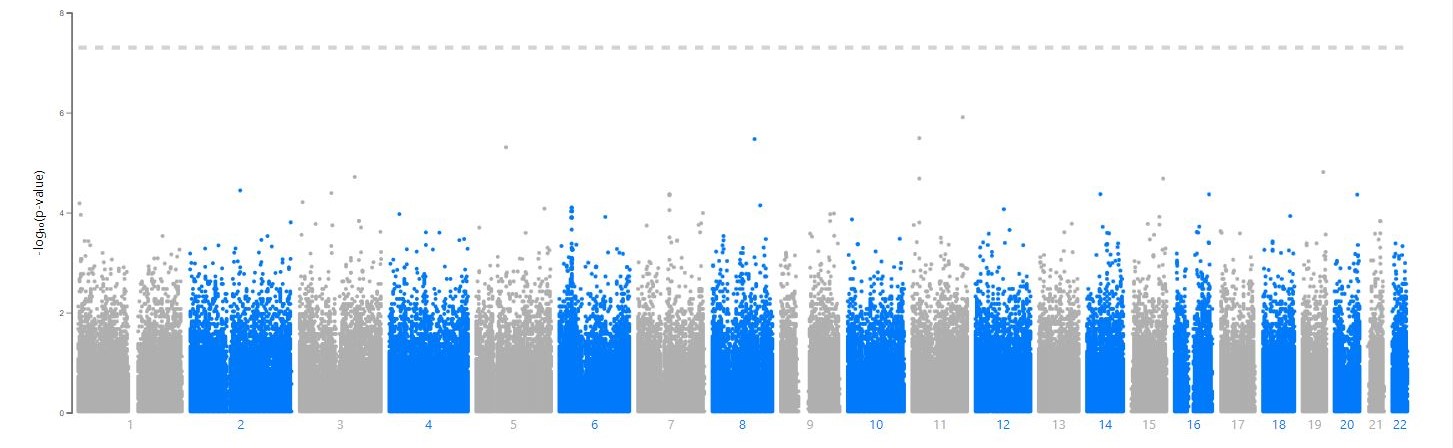


**Supplementary Figure 1. Outercanthal Width and GWAS association results.** The Manhattan plot shows the SNP associations to the measurement represented by the (-log_10_[P]) on the (y-axis) and the genotyped SNPs (each dot represents a SNP according to the chromosomal position (numbered from 1-22) on the (x-axis). The horizontal dashed grey line shows the genome-wide significance threshold which is (*p* = 5.0×10^−8^).


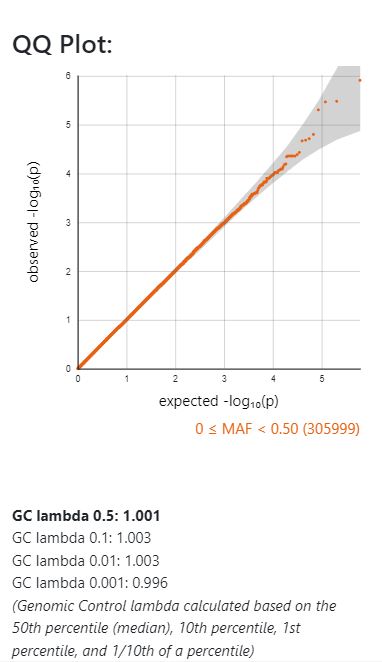


**Supplementary Figure 2. Q-Q Plot for the GWAS of Outercanthal Width measurement.** GC lambda 0.5: 1.001.


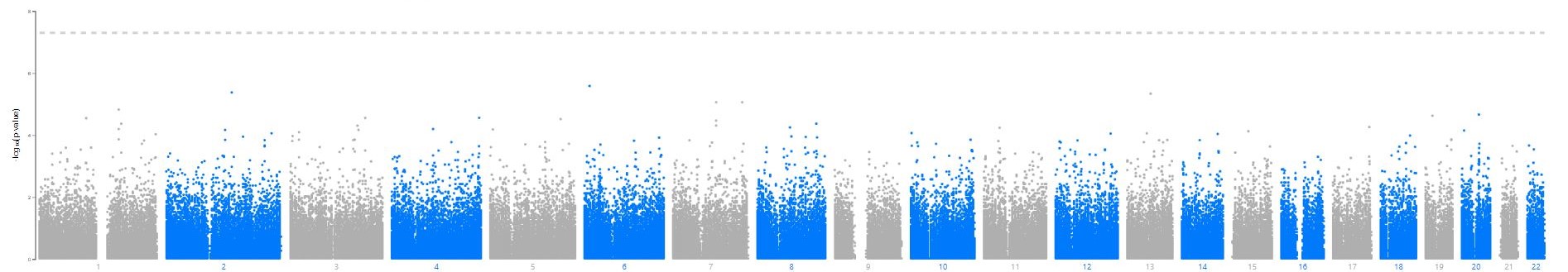


**Supplementary Figure 3. Innercanthal width and GWAS association results.** The Manhattan plot shows the SNP associations to the measurement represented by the (-log_10_[P]) on the (y-axis) and the genotyped SNPs (each dot represents a SNP according to the chromosomal position (numbered from 1-22) on the (x-axis). The horizontal dashed grey line shows the genome-wide significance threshold which is (*p* = 5.0×10^−8^).


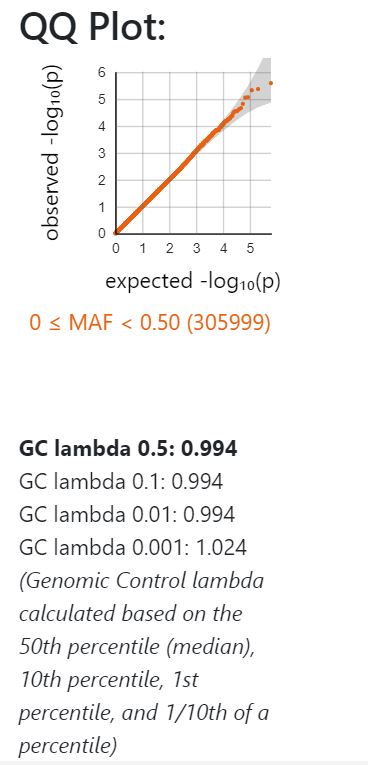


**Supplementary Figure 4. Q-Q Plot for the GWAS of Innercanthal width measurement.** GC lambda 0.5: 0.994.


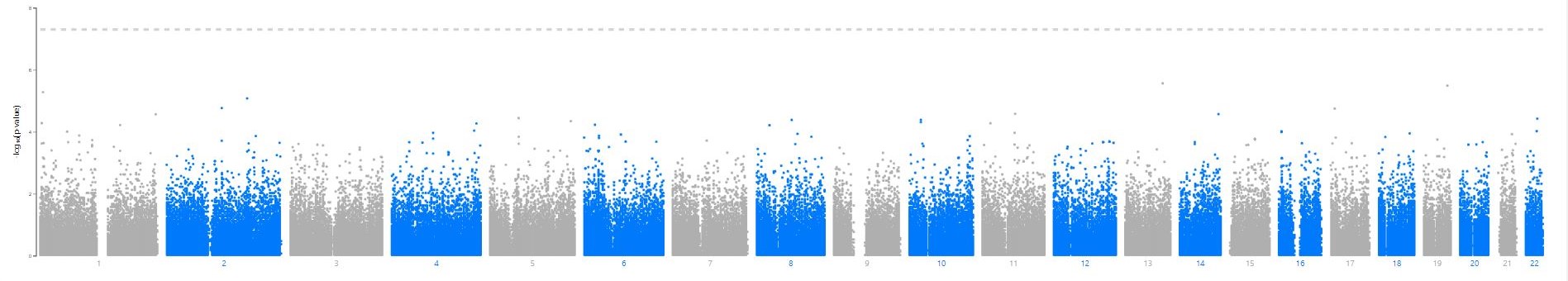


**Supplementary Figure 5. Left Palpebral Fissure Length and GWAS association results.** The Manhattan plot shows the SNP associations to the measurement represented by the (-log_10_[P]) on the (y-axis) and the genotyped SNPs (each dot represents a SNP according to the chromosomal position (numbered from 1-22) on the (x-axis). The horizontal dashed grey line shows the genome-wide significance threshold which is (*p* = 5.0×10^−8^).


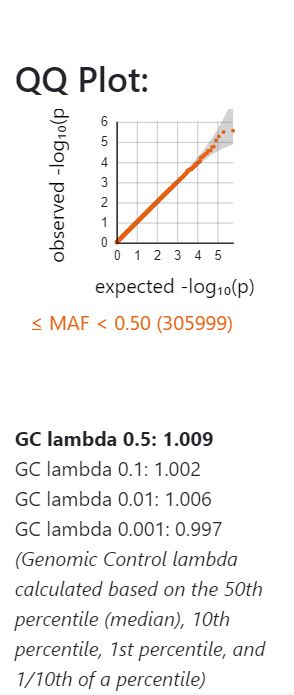


**Supplementary Figure 6. Q-Q Plot for the GWAS of Left Palpebral Fissure Length measurement.** GC lambda 0.5: 1.009.


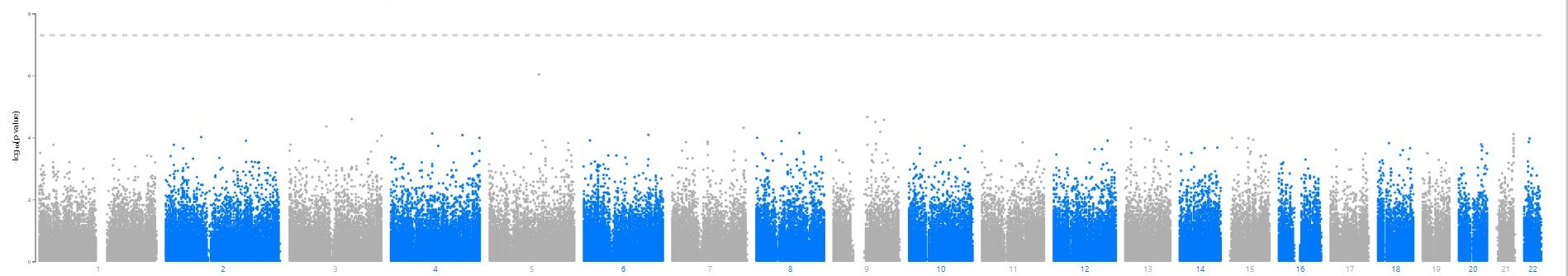


**Supplementary Figure 7. Right Palpebral Fissure Length and GWAS association results.** The Manhattan plot shows the SNP associations to the measurement represented by the (-log_10_[P]) on the (y-axis) and the genotyped SNPs (each dot represents a SNP according to the chromosomal position (numbered from 1-22) on the (x-axis). The horizontal dashed grey line shows the genome-wide significance threshold which is (*p* = 5.0×10^−8^).


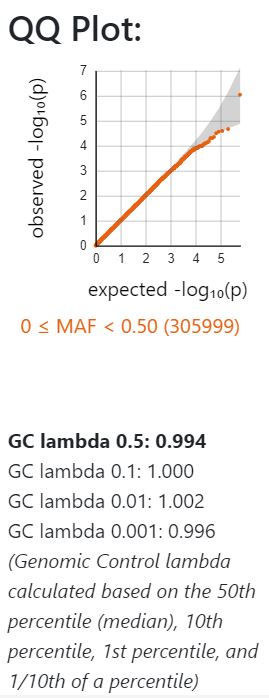


**Supplementary Figure 8. Q-Q Plot for the GWAS of Right Palpebral Fissure Length measurement.** GC lambda 0.5: 0.994.


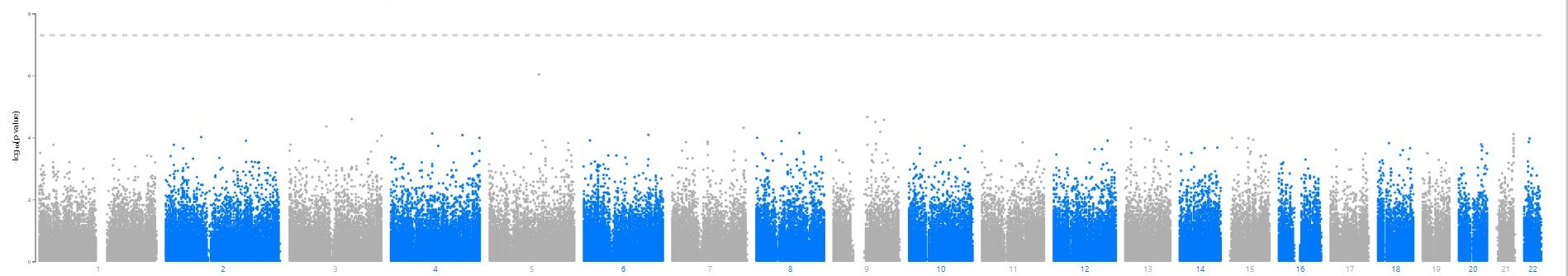


**Supplementary Figure 9. Facial Height and GWAS association results.** The Manhattan plot shows the SNP associations to the measurement represented by the (-log_10_[P]) on the (y-axis) and the genotyped SNPs (each dot represents a SNP according to the chromosomal position (numbered from 1-22) on the (x-axis). The horizontal dashed grey line shows the genome-wide significance threshold which is (*p* = 5.0×10^−8^).


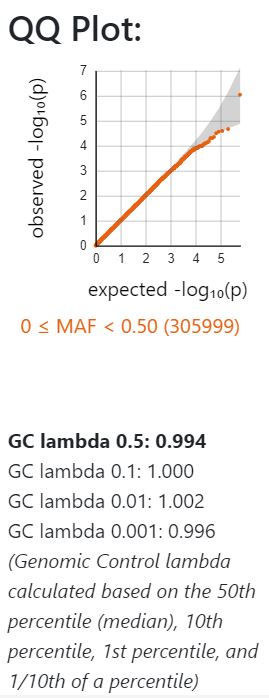


**Supplementary Figure 10. Q-Q Plot for the GWAS of Facial Height measurement.** GC lambda 0.5: 0.994.


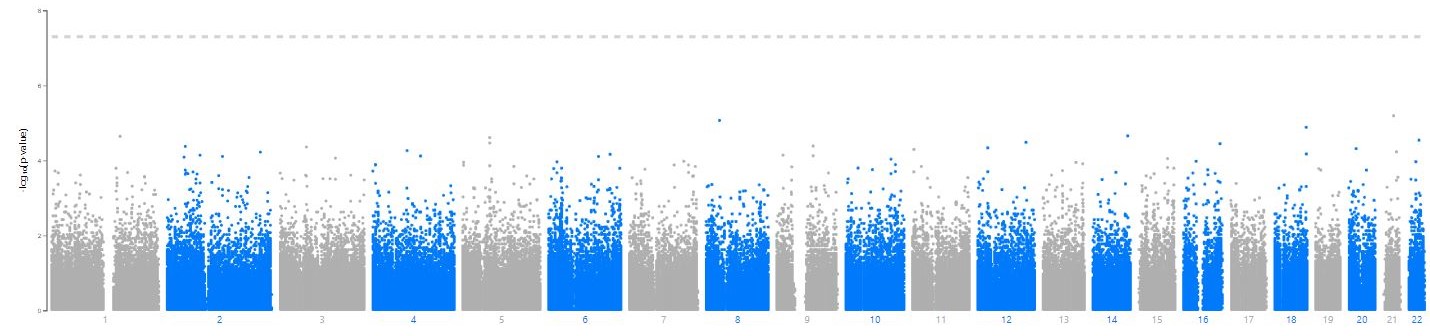


**Supplementary Figure 11. Bizygomatic Width and GWAS association results.** The Manhattan plot shows the SNP associations to the measurement represented by the (-log_10_[P]) on the (y-axis) and the genotyped SNPs (each dot represents a SNP according to the chromosomal position (numbered from 1-22) on the (x-axis). The horizontal dashed grey line shows the genome-wide significance threshold which is (*p* = 5.0×10^−8^).


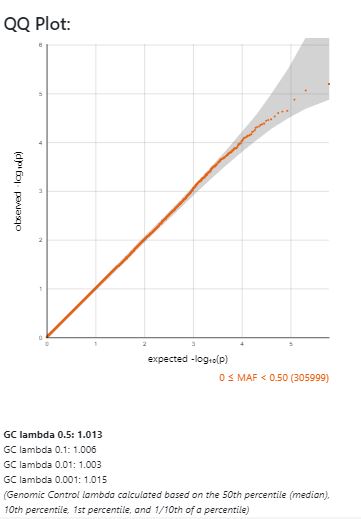


**Supplementary Figure 12. Q-Q Plot for the GWAS of Bizygomatic Width measurement.** GC lambda 0.5: 1.013.


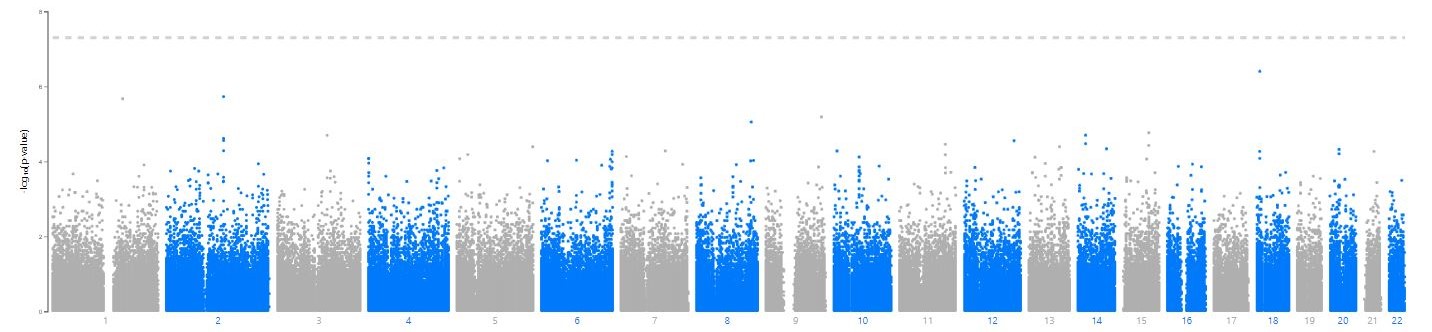


**Supplementary Figure 13. Mandibular Width and GWAS association results.** The Manhattan plot shows the SNP associations to the measurement represented by the (-log_10_[P]) on the (y-axis) and the genotyped SNPs (each dot represents a SNP according to the chromosomal position (numbered from 1-22) on the (x-axis). The horizontal dashed grey line shows the genome-wide significance threshold which is (*p* = 5.0×10^−8^).


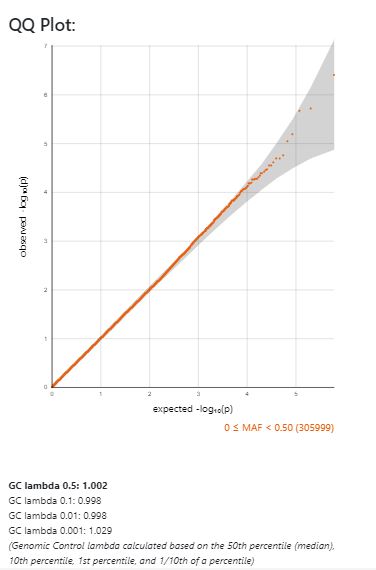


**Supplementary Figure 14. Q-Q Plot for the GWAS of Mandibular Width measurement.** GC lambda 0.5: 1.002.


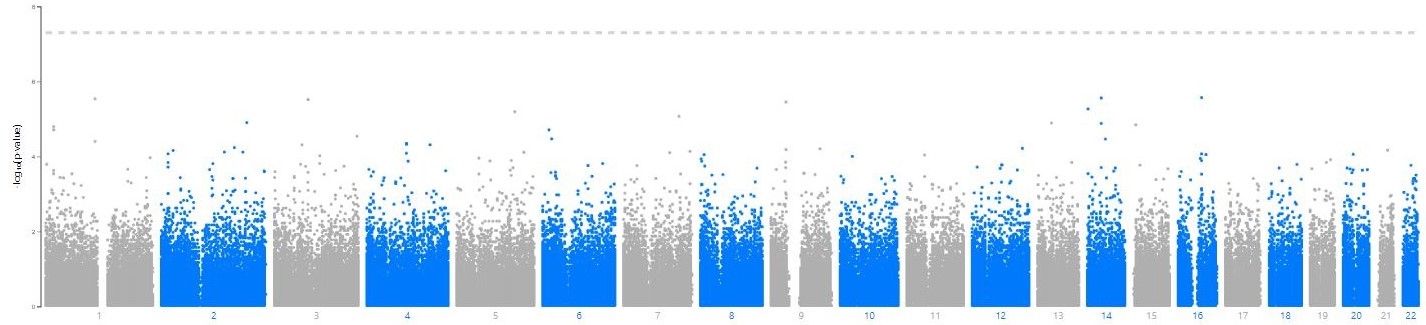


**Supplementary Figure 15. Bifrontal Width and GWAS association results.** The Manhattan plot shows the SNP associations to the measurement represented by the (-log_10_[P]) on the (y-axis) and the genotyped SNPs (each dot represents a SNP according to the chromosomal position (numbered from 1-22) on the (x-axis). The horizontal dashed grey line shows the genome-wide significance threshold which is (*p* = 5.0×10^−8^).


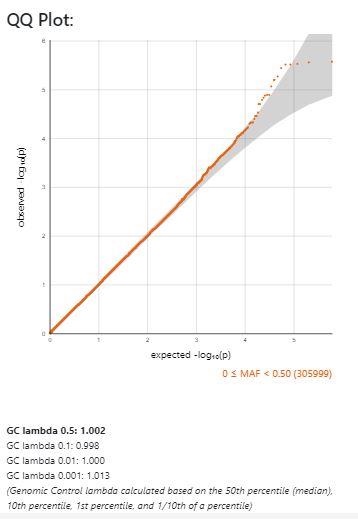


**Supplementary Figure 16. Q-Q Plot for the GWAS of Bifrontal Width measurement.** GC lambda 0.5: 1.002.


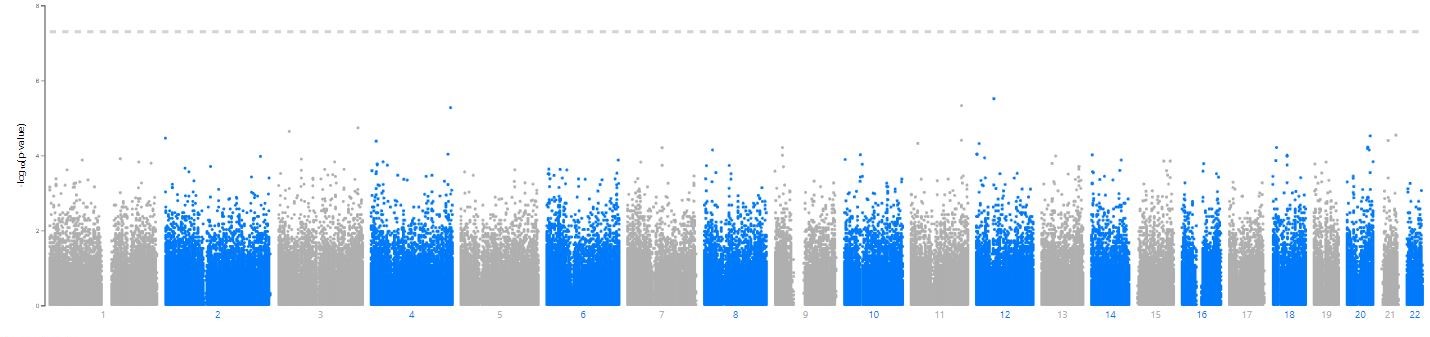


**Supplementary Figure 17. Lower Vermilion Height and GWAS association results.** The Manhattan plot shows the SNP associations to the measurement represented by the (-log_10_[P]) on the (y-axis) and the genotyped SNPs (each dot represents a SNP according to the chromosomal position (numbered from 1-22) on the (x-axis). The horizontal dashed grey line shows the genome-wide significance threshold which is (*p* = 5.0×10^−8^).


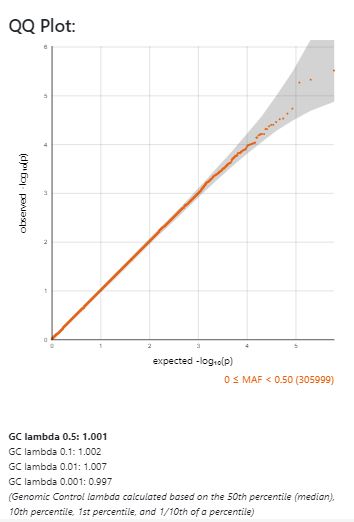


**Supplementary Figure 18. Q-Q Plot for the GWAS of Lower Vermilion Height measurement.** GC lambda 0.5: 1.001.


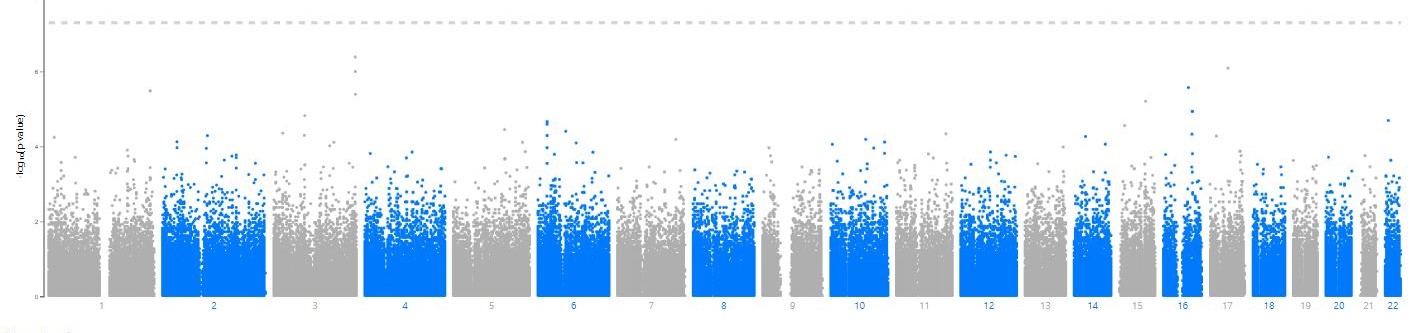


**Supplementary Figure 19. Philtral Length and GWAS association results.** The Manhattan plot shows the SNP associations to the measurement represented by the (-log_10_[P]) on the (y-axis) and the genotyped SNPs (each dot represents a SNP according to the chromosomal position (numbered from 1-22) on the (x-axis). The horizontal dashed grey line shows the genome-wide significance threshold which is (*p* = 5.0×10^−8^).


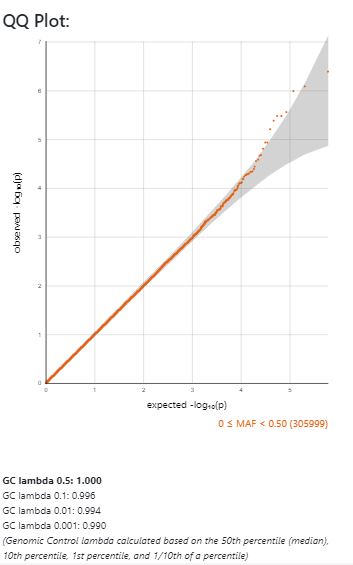


**Supplementary Figure 20. Q-Q Plot for the GWAS of Philtral Length measurement.** GC lambda 0.5: 1.000.


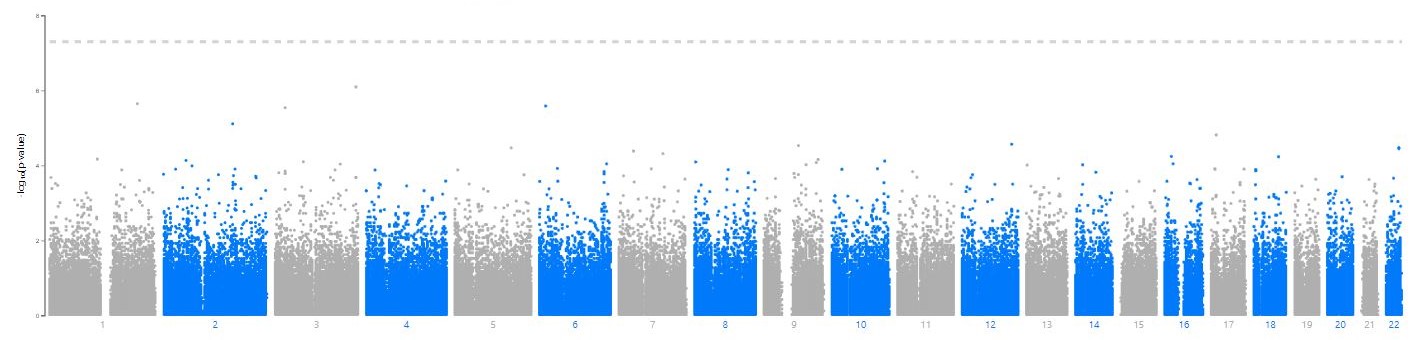


**Supplementary Figure 21. Labial Fissure Width and GWAS association results.** The Manhattan plot shows the SNP associations to the measurement represented by the (-log_10_[P]) on the (y-axis) and the genotyped SNPs (each dot represents a SNP according to the chromosomal position (numbered from 1-22) on the (x-axis). The horizontal dashed grey line shows the genome-wide significance threshold which is (*p* = 5.0×10^−8^).


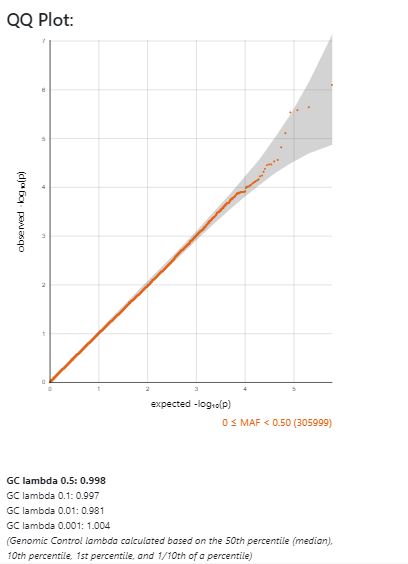


**Supplementary Figure 22. Q-Q Plot for the GWAS of Labial Fissure Width measurement.** GC lambda 0.5: 0.998.


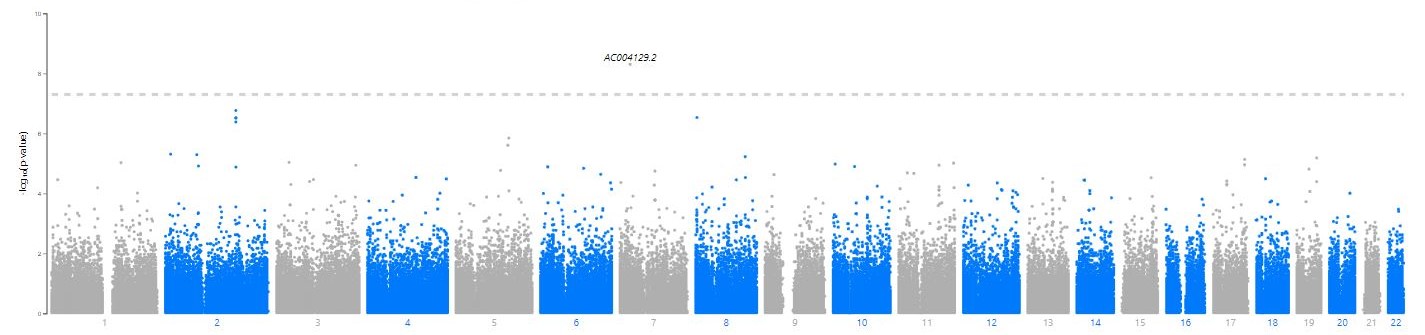


**Supplementary Figure 23. Philtral Width and GWAS association results.** The Manhattan plot shows the SNP associations to the measurement represented by the (-log_10_[P]) on the (y-axis) and the genotyped SNPs (each dot represents a SNP according to the chromosomal position (numbered from 1-22) on the (x-axis). The horizontal dashed grey line shows the genome-wide significance threshold which is (*p* = 5.0×10^−8^).


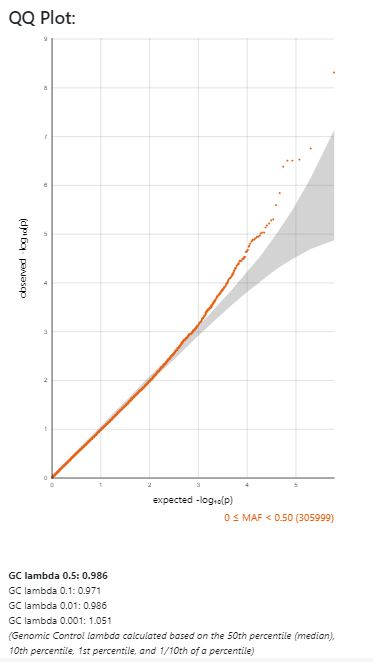


**Supplementary Figure 24. Q-Q Plot for the GWAS of Philtral Width measurement.** GC lambda 0.5: 0.986.


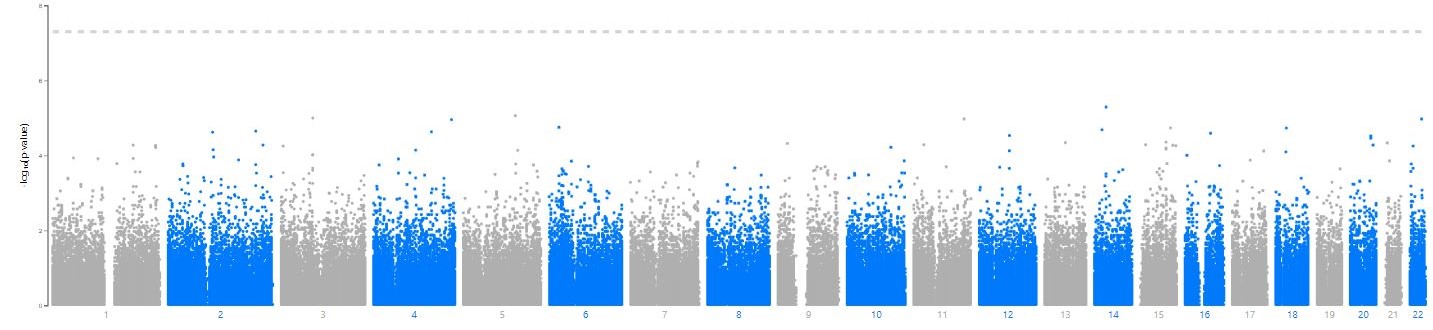


**Supplementary Figure 25. Upper Lip Height and GWAS association results.** The Manhattan plot shows the SNP associations to the measurement represented by the (-log_10_[P]) on the (y-axis) and the genotyped SNPs (each dot represents a SNP according to the chromosomal position (numbered from 1-22) on the (x-axis). The horizontal dashed grey line shows the genome-wide significance threshold which is (*p* = 5.0×10^−8^).


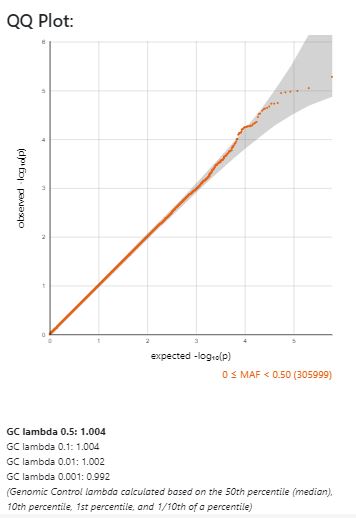


**Supplementary Figure 26. Q-Q Plot for the GWAS of Upper Lip Height measurement. GC lambda 0.5: 1.004.**


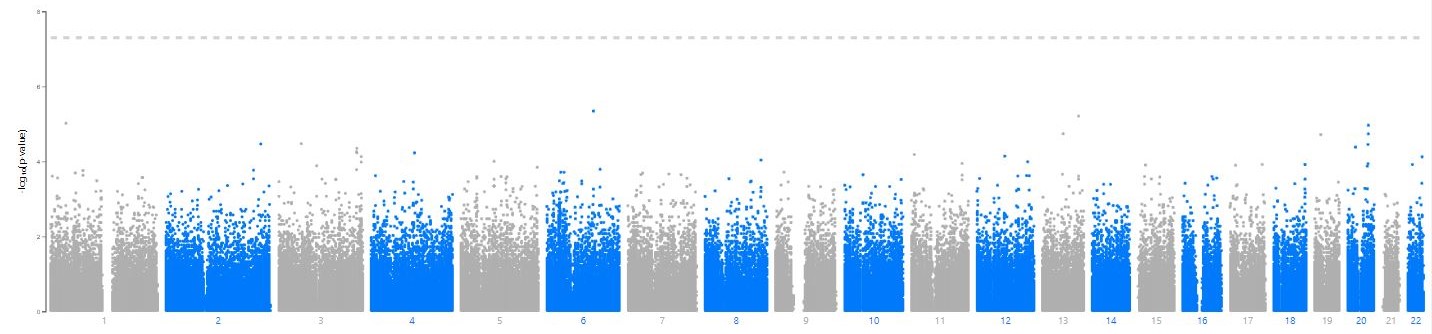


**Supplementary Figure 27. Upper Vermilion Height and GWAS association results.** The Manhattan plot shows the SNP associations to the measurement represented by the (-log_10_[P]) on the (y-axis) and the genotyped SNPs (each dot represents a SNP according to the chromosomal position (numbered from 1-22) on the (x-axis). The horizontal dashed grey line shows the genome-wide significance threshold which is (*p* = 5.0×10^−8^).


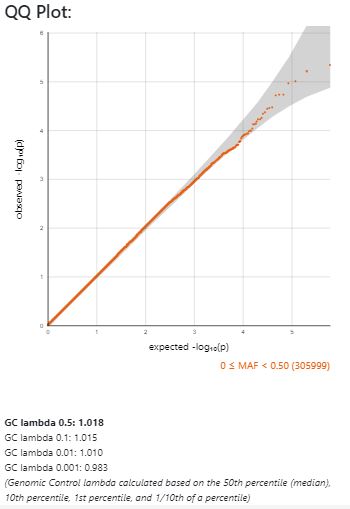


**Supplementary Figure 28. Q-Q Plot for the GWAS of Upper Vermilion Height measurement.** GC lambda 0.5: 1.018.


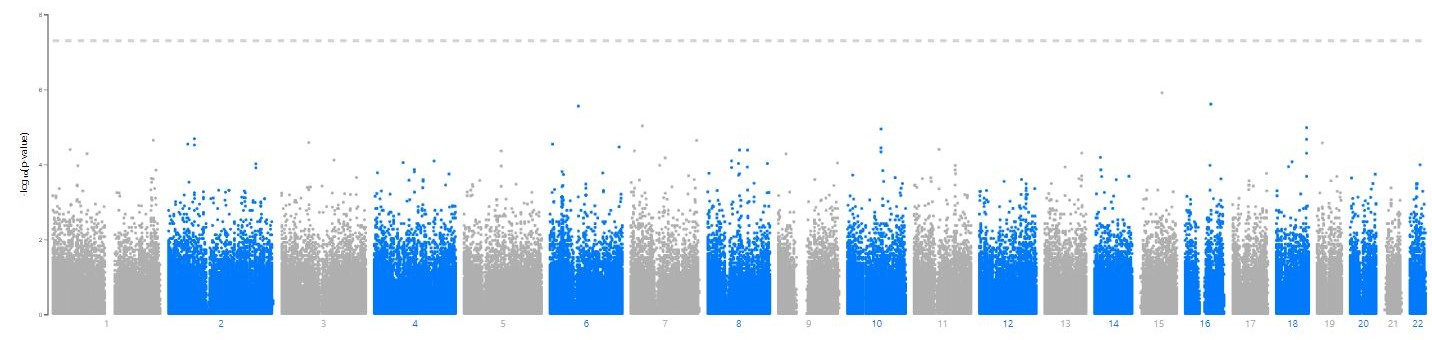


**Supplementary Figure 29. Subnasal Width and GWAS association results.** The Manhattan plot shows the SNP associations to the measurement represented by the (-log_10_[P]) on the (y-axis) and the genotyped SNPs (each dot represents a SNP according to the chromosomal position (numbered from 1-22) on the (x-axis). The horizontal dashed grey line shows the genome-wide significance threshold which is (*p* = 5.0×10^−8^).


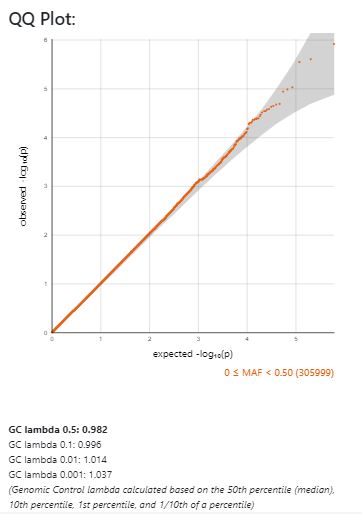


**Supplementary Figure 30. Q-Q Plot for the GWAS of Subnasal Width measurement.** GC lambda 0.5: 0.982.


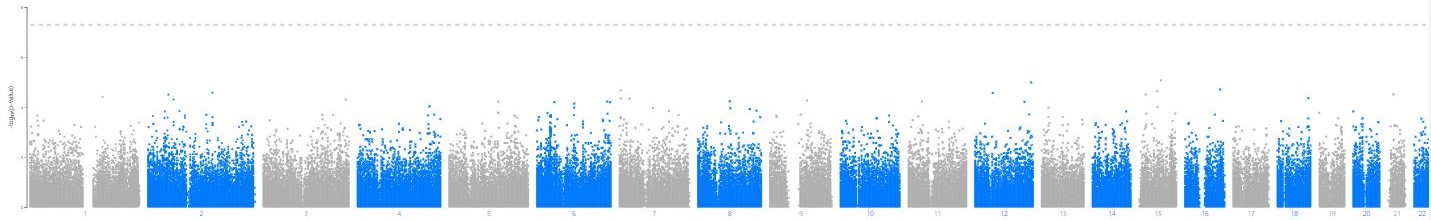


**Supplementary Figure 31. Nasal Width and GWAS association results.** The Manhattan plot shows the SNP associations to the measurement represented by the (-log_10_[P]) on the (y-axis) and the genotyped SNPs (each dot represents a SNP according to the chromosomal position (numbered from 1-22) on the (x-axis). The horizontal dashed grey line shows the genome-wide significance threshold which is (*p* = 5.0×10^−8^).


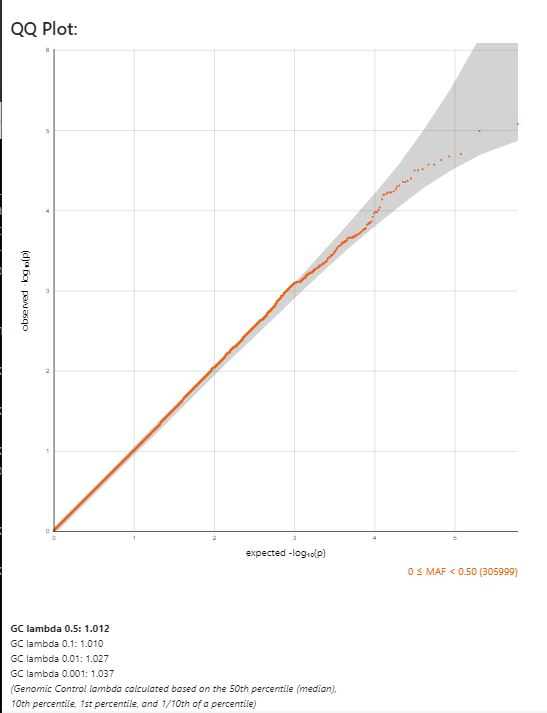


**Supplementary Figure 32. Q-Q Plot for the GWAS of Nasal Width measurement.** GC lambda 0.5: 1.012.


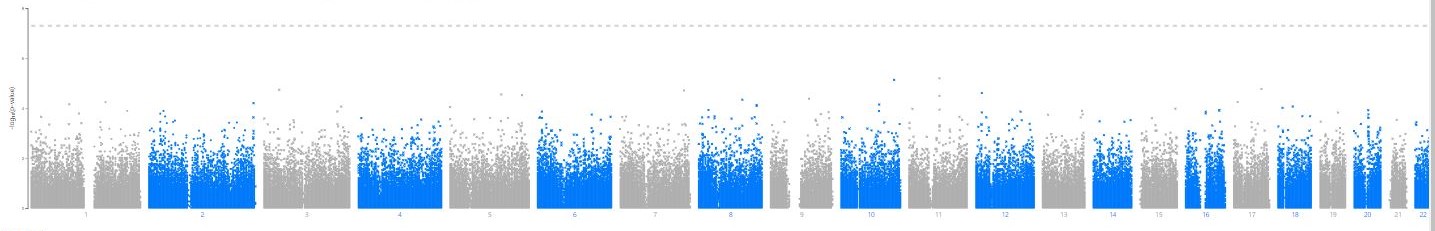


**Supplementary Figure 33. Columella Length and GWAS association results.** The Manhattan plot shows the SNP associations to the measurement represented by the (-log_10_[P]) on the (y-axis) and the genotyped SNPs (each dot represents a SNP according to the chromosomal position (numbered from 1-22) on the (x-axis). The horizontal dashed grey line shows the genome-wide significance threshold which is (*p* = 5.0×10^−8^).


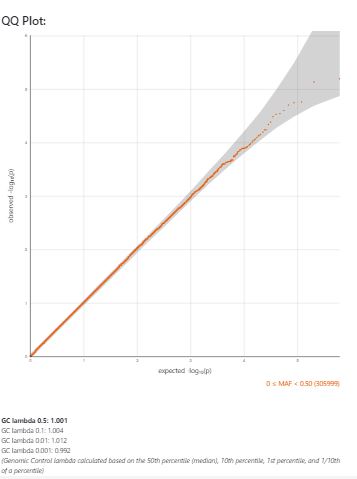


**Supplementary Figure 34. Q-Q Plot for the GWAS of Columella Length measurement.** GC lambda 0.5: 1.001.


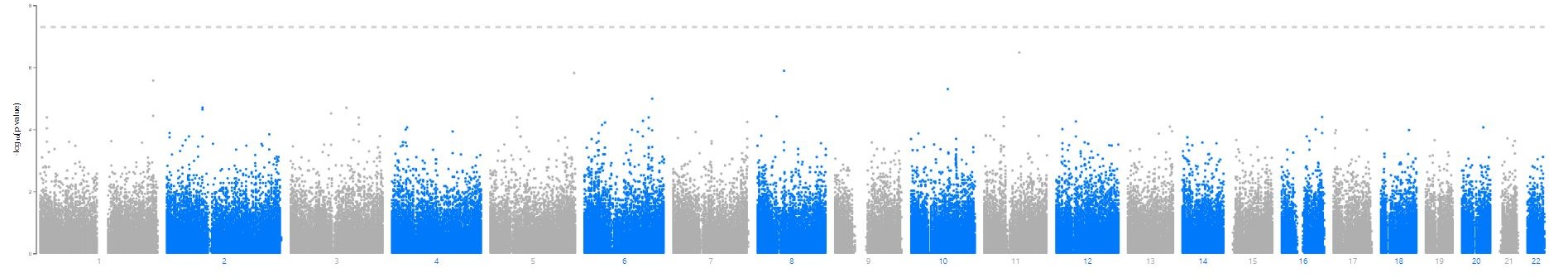


**Supplementary Figure 35. Nasal Bridge Length and GWAS association results.** The Manhattan plot shows the SNP associations to the measurement represented by the (-log_10_[P]) on the (y-axis) and the genotyped SNPs (each dot represents a SNP according to the chromosomal position (numbered from 1-22) on the (x-axis). The horizontal dashed grey line shows the genome-wide significance threshold which is (*p* = 5.0×10^−8^).


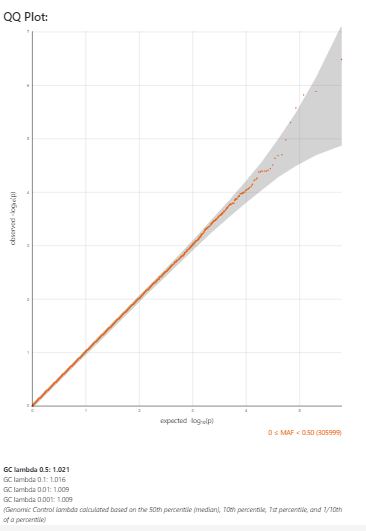


**Supplementary Figure 36. Q-Q Plot for the GWAS of Nasal Bridge Length measurement.** GC lambda 0.5: 1.021.


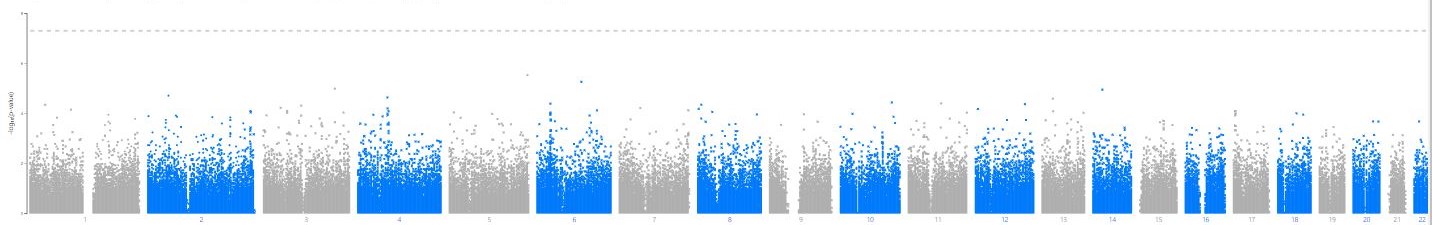


**Supplementary Figure 37. Nasal Height and GWAS association results.** The Manhattan plot shows the SNP associations to the measurement represented by the (-log_10_[P]) on the (y-axis) and the genotyped SNPs (each dot represents a SNP according to the chromosomal position (numbered from 1-22) on the (x-axis). The horizontal dashed grey line shows the genome-wide significance threshold which is (*p* = 5.0×10^−8^).


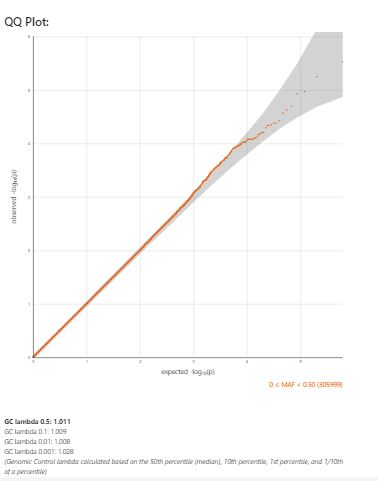


**Supplementary Figure 38. Q-Q Plot for the GWAS of Nasal Height measurement.** GC lambda 0.5: 1.011.


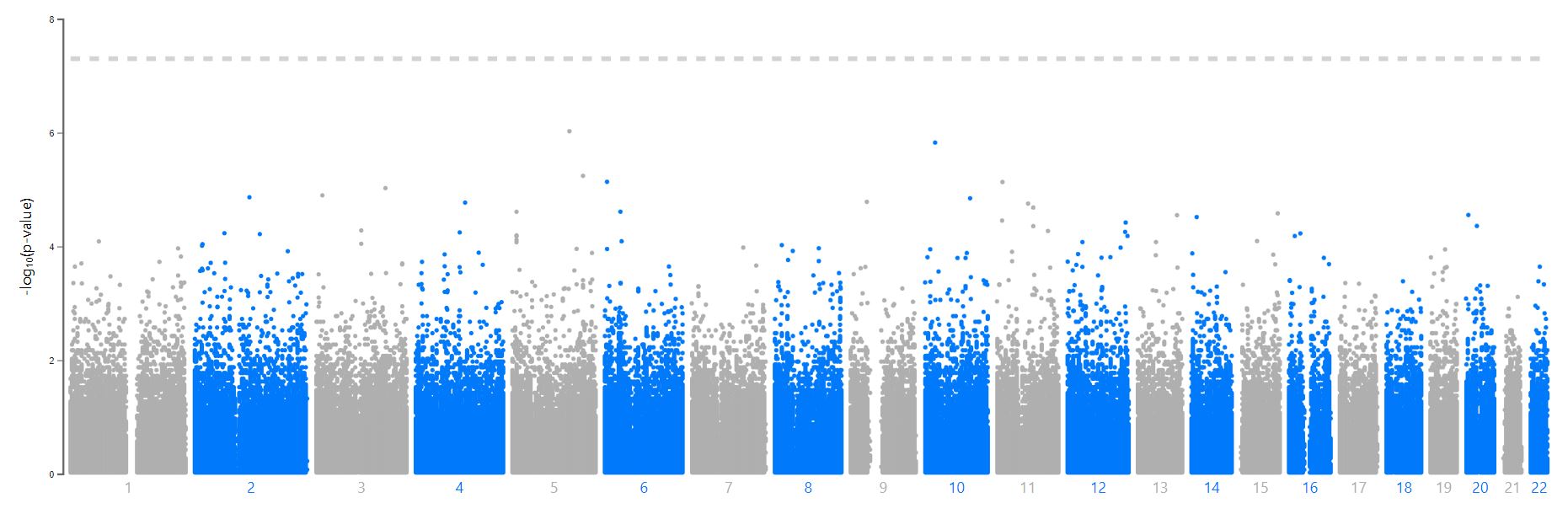


**Supplementary Figure 39. Nasal Root Width and GWAS association results.** The Manhattan plot shows the SNP associations to the measurement represented by the (-log_10_[P]) on the (y-axis) and the genotyped SNPs (each dot represents a SNP according to the chromosomal position (numbered from 1-22) on the (x-axis). The horizontal dashed grey line shows the genome-wide significance threshold which is (*p* = 5.0×10^−8^).


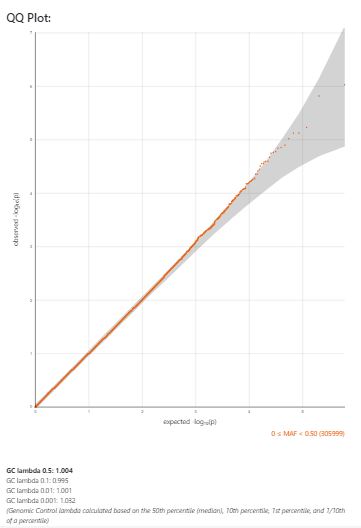


**Supplementary Figure 40. Q-Q Plot for the GWAS of Nasal Root Width measurement.** GC lambda 0.5: 1.004.


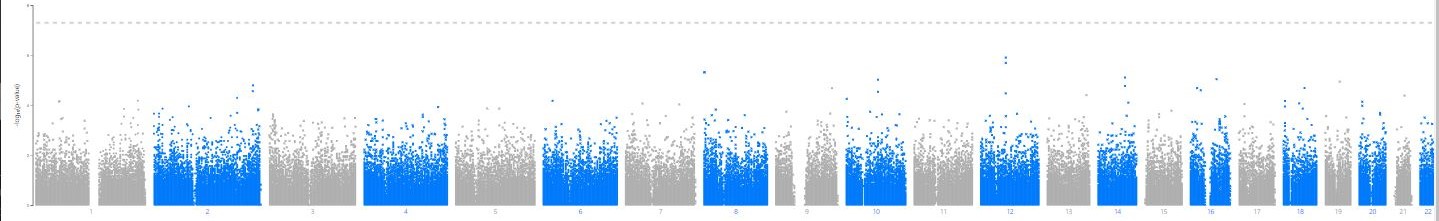


**Supplementary Figure 41. Interpupillary Distance and GWAS association results.** The Manhattan plot shows the SNP associations to the measurement represented by the (-log_10_[P]) on the (y-axis) and the genotyped SNPs (each dot represents a SNP according to the chromosomal position (numbered from 1-22) on the (x-axis). The horizontal dashed grey line shows the genome-wide significance threshold which is (*p* = 5.0×10^−8^).


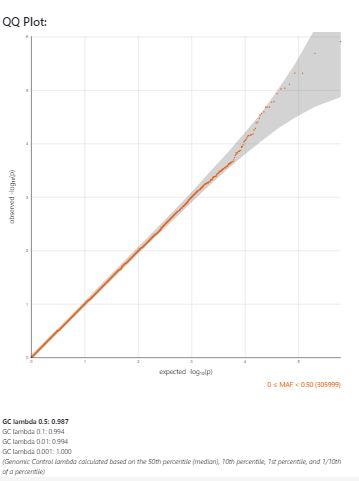


**Supplementary Figure 42. Q-Q Plot for the GWAS of Interpupillary Distance measurement.** GC lambda 0.5: 0.987.


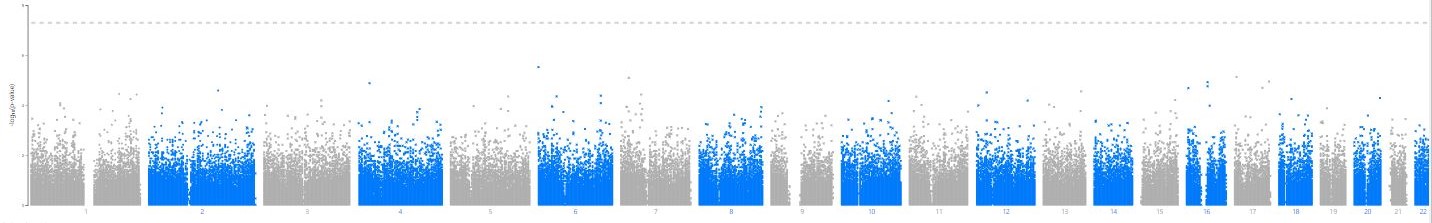


**Supplementary Figure 43. Left Palpebral Fissure Inclination and GWAS association results.** The Manhattan plot shows the SNP associations to the measurement represented by the (-log_10_[P]) on the (y-axis) and the genotyped SNPs (each dot represents a SNP according to the chromosomal position (numbered from 1-22) on the (x-axis). The horizontal dashed grey line shows the genome-wide significance threshold which is (*p* = 5.0×10^−8^).


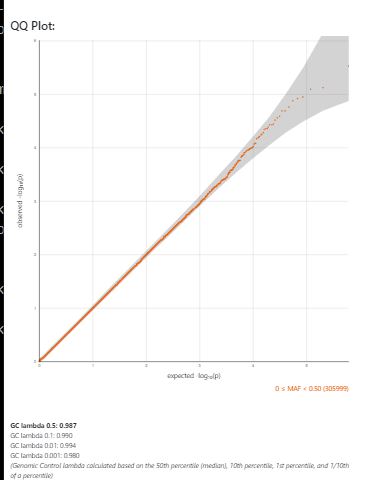


**Supplementary Figure 44. Q-Q Plot for the GWAS of Left Palpebral Fissure Inclination measurement.** GC lambda 0.5: 0.987.


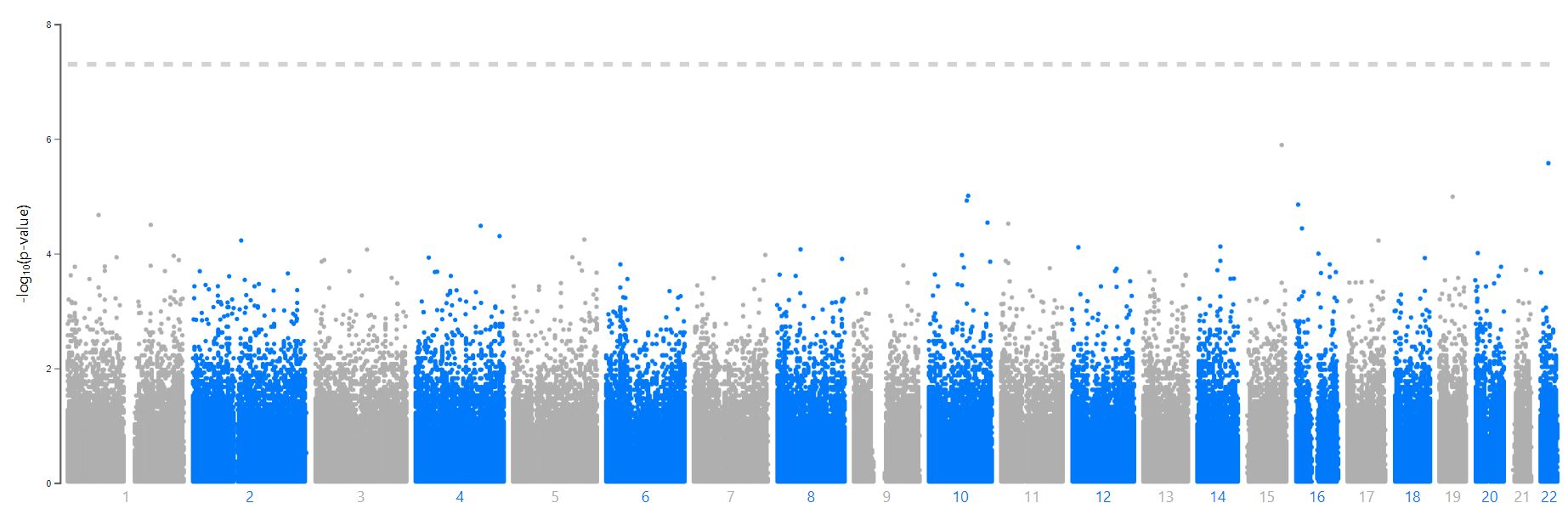


**Supplementary Figure 45. Right Palpebral Fissure Inclination and GWAS association results.** The Manhattan plot shows the SNP associations to the measurement represented by the (-log_10_[P]) on the (y-axis) and the genotyped SNPs (each dot represents a SNP according to the chromosomal position (numbered from 1-22) on the (x-axis). The horizontal dashed grey line shows the genome-wide significance threshold which is (*p* = 5.0×10^−8^).


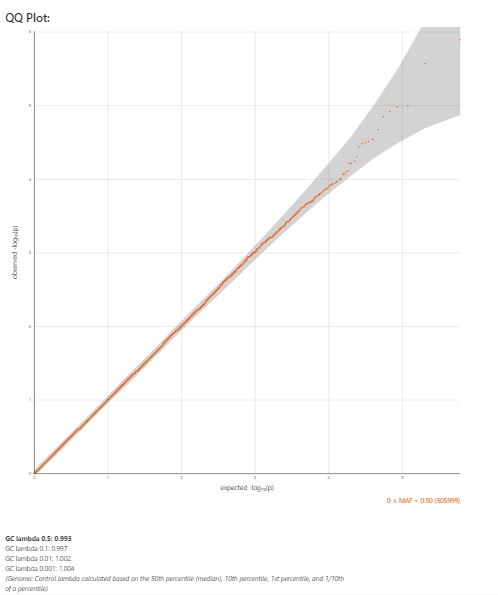


**Supplementary Figure 46. Q-Q Plot for the GWAS of Right Palpebral Fissure Inclination measurement.** GC lambda 0.5: 0.993.


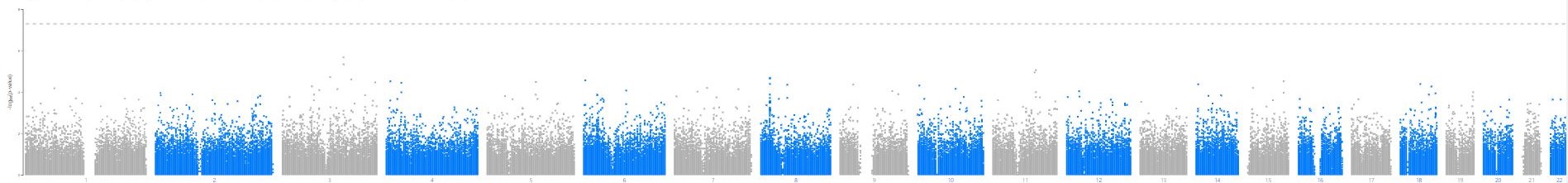


**Supplementary Figure 47. Left Palpebral Fissure Height and GWAS association results.** The Manhattan plot shows the SNP associations to the measurement represented by the (-log_10_[P]) on the (y-axis) and the genotyped SNPs (each dot represents a SNP according to the chromosomal position (numbered from 1-22) on the (x-axis). The horizontal dashed grey line shows the genome-wide significance threshold which is (*p* = 5.0×10^−8^).


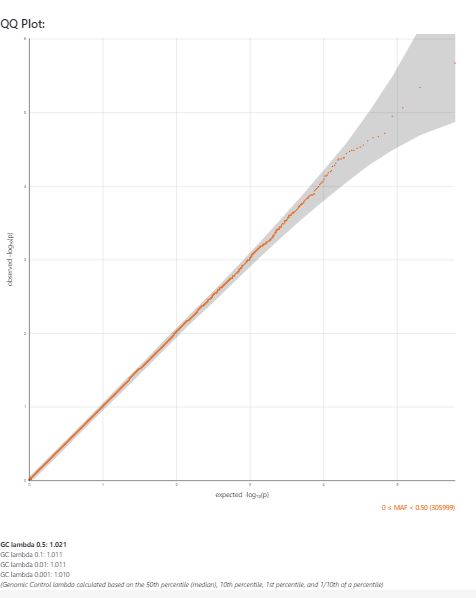


**Supplementary Figure 48. Q-Q Plot for the GWAS of Left Palpebral Fissure Height measurement.** GC lambda 0.5: 1.021.


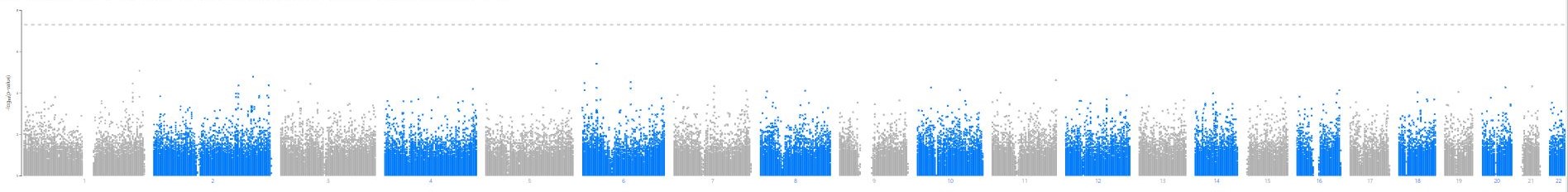


**Supplementary Figure 49. Right Palpebral Fissure Height and GWAS association results.** The Manhattan plot shows the SNP associations to the measurement represented by the (-log_10_[P]) on the (y-axis) and the genotyped SNPs (each dot represents a SNP according to the chromosomal position (numbered from 1-22) on the (x-axis). The horizontal dashed grey line shows the genome-wide significance threshold which is (*p* = 5.0×10^−8^).


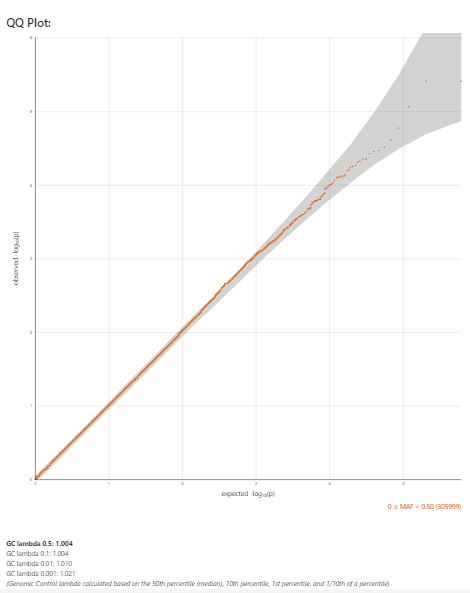


**Supplementary Figure 50. Q-Q Plot for the GWAS of Right Palpebral Fissure Height measurement.** GC lambda 0.5: 1.004.


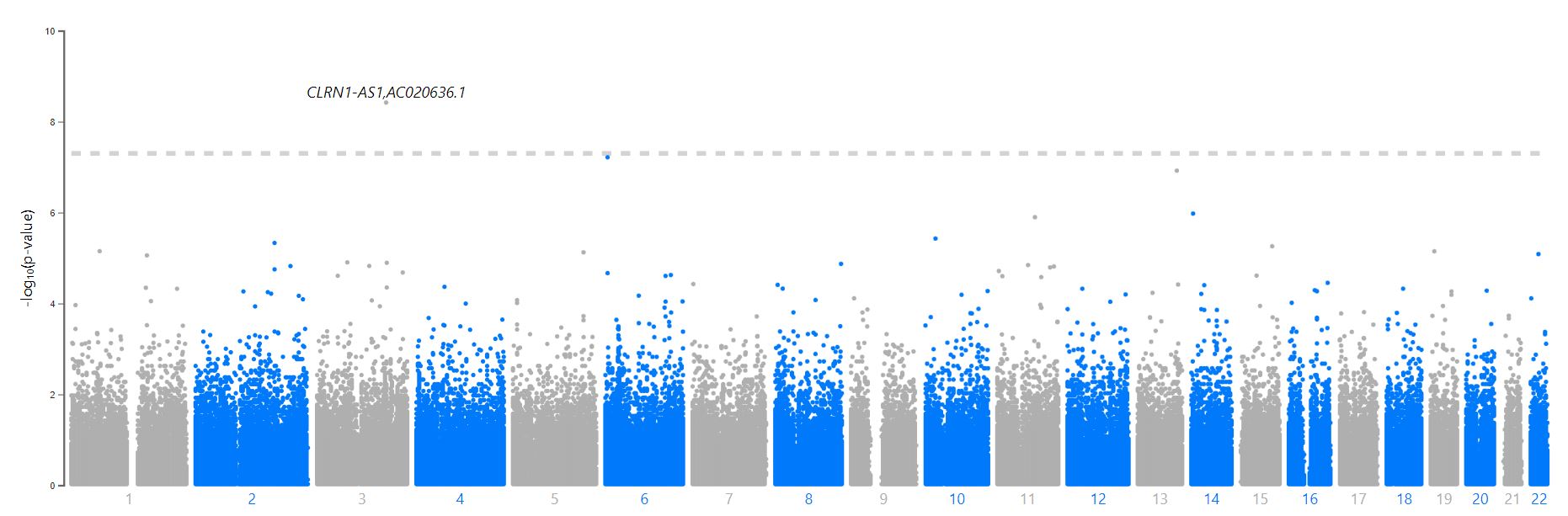


**Supplementary Figure 51. Mandibular Contour and GWAS association results.** The Manhattan plot shows the SNP associations to the measurement represented by the (-log_10_[P]) on the (y-axis) and the genotyped SNPs (each dot represents a SNP according to the chromosomal position (numbered from 1-22) on the (x-axis). The horizontal dashed grey line shows the genome-wide significance threshold which is (*p* = 5.0×10^−8^).


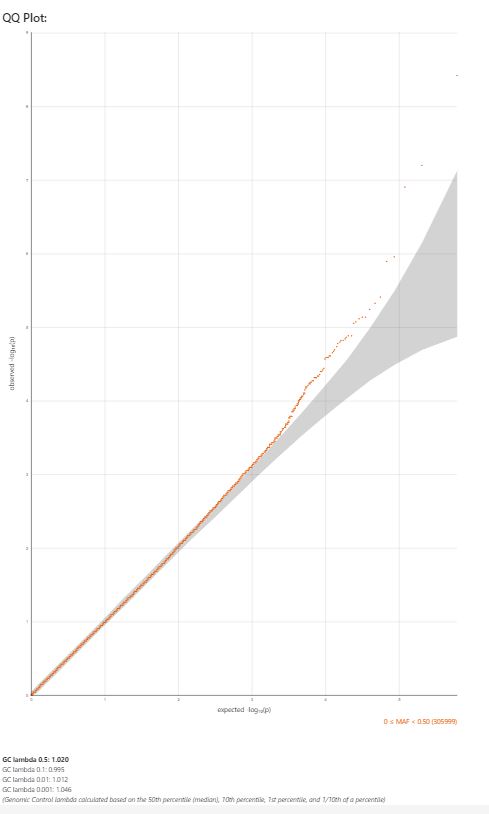


**Supplementary Figure 52. Q-Q Plot for the GWAS of Mandibular Contour measurement.** GC lambda 0.5: 1.020.


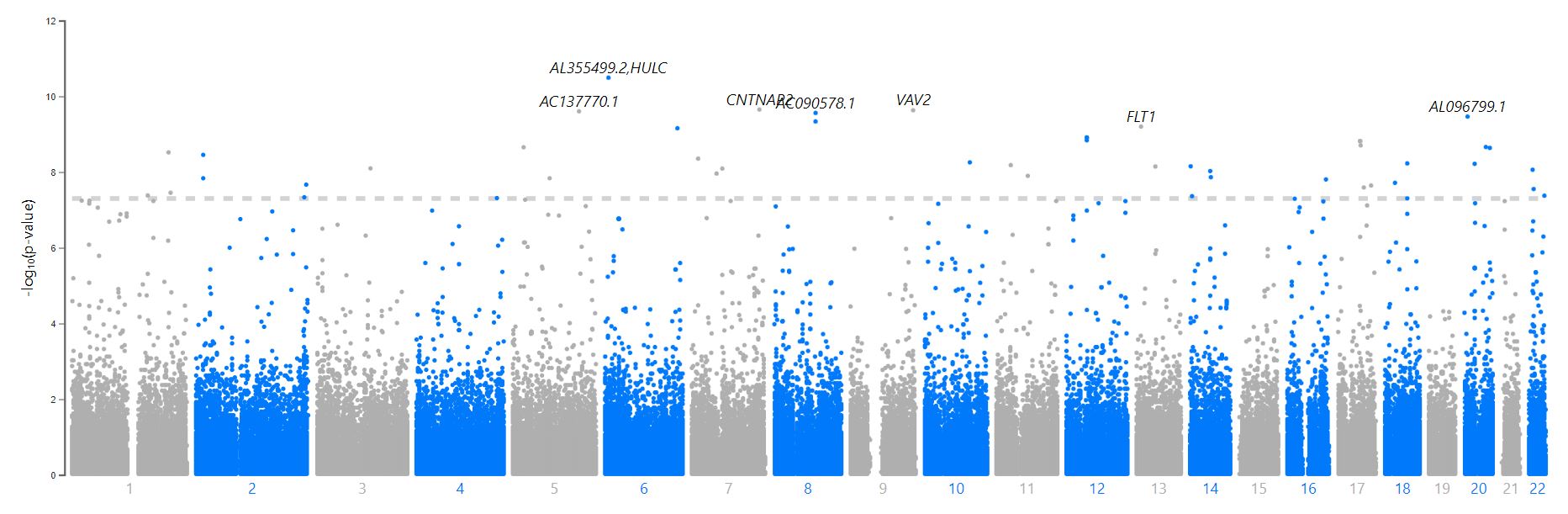


**Supplementary Figure 53. Facial Convexity and GWAS association results.** The Manhattan plot shows the SNP associations to the measurement represented by the (-log_10_[P]) on the (y-axis) and the genotyped SNPs (each dot represents a SNP according to the chromosomal position (numbered from 1-22) on the (x-axis). The horizontal dashed grey line shows the genome-wide significance threshold which is (*p* = 5.0×10^−8^).


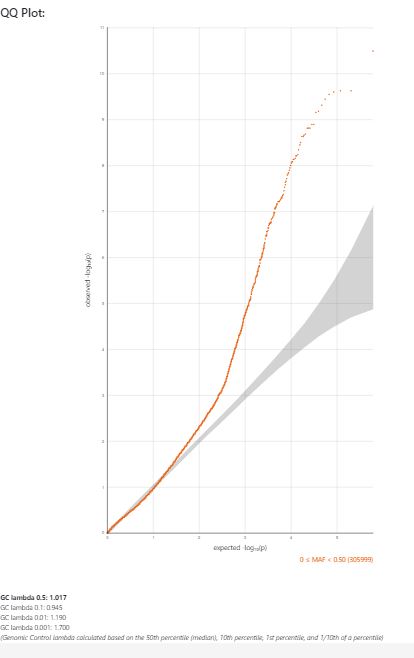


**Supplementary Figure 54. Q-Q Plot for the GWAS of Facial Convexity measurement.** GC lambda 0.5: 1.017.


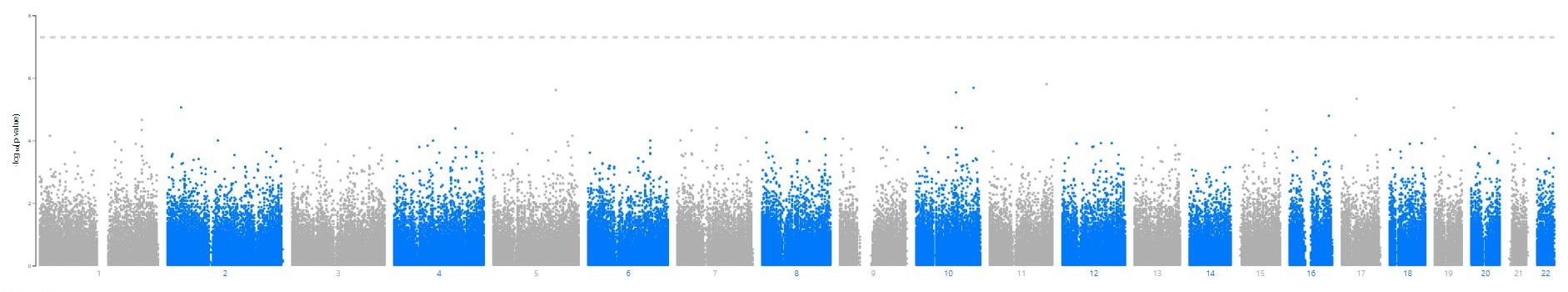


**Supplementary Figure 55. Nasal Protrusion and GWAS association results.** The Manhattan plot shows the SNP associations to the measurement represented by the (-log_10_[P]) on the (y-axis) and the genotyped SNPs (each dot represents a SNP according to the chromosomal position (numbered from 1-22) on the (x-axis). The horizontal dashed grey line shows the genome-wide significance threshold which is (*p* = 5.0×10^−8^).


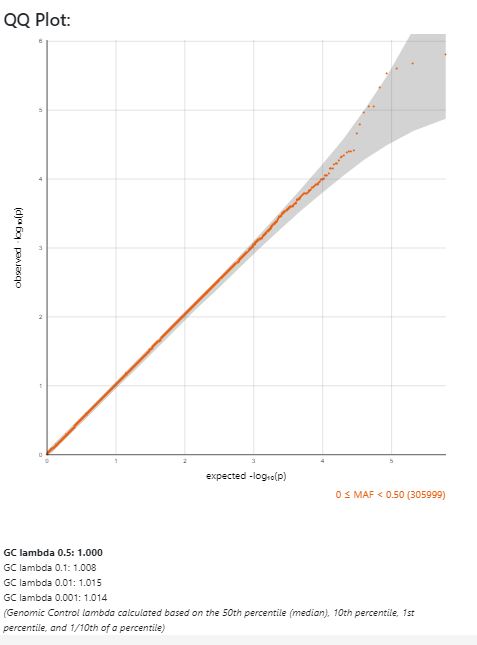


**Supplementary Figure 56. Q-Q Plot for the GWAS of Nasal Protrusion measurement.** GC lambda 0.5: 1.000.


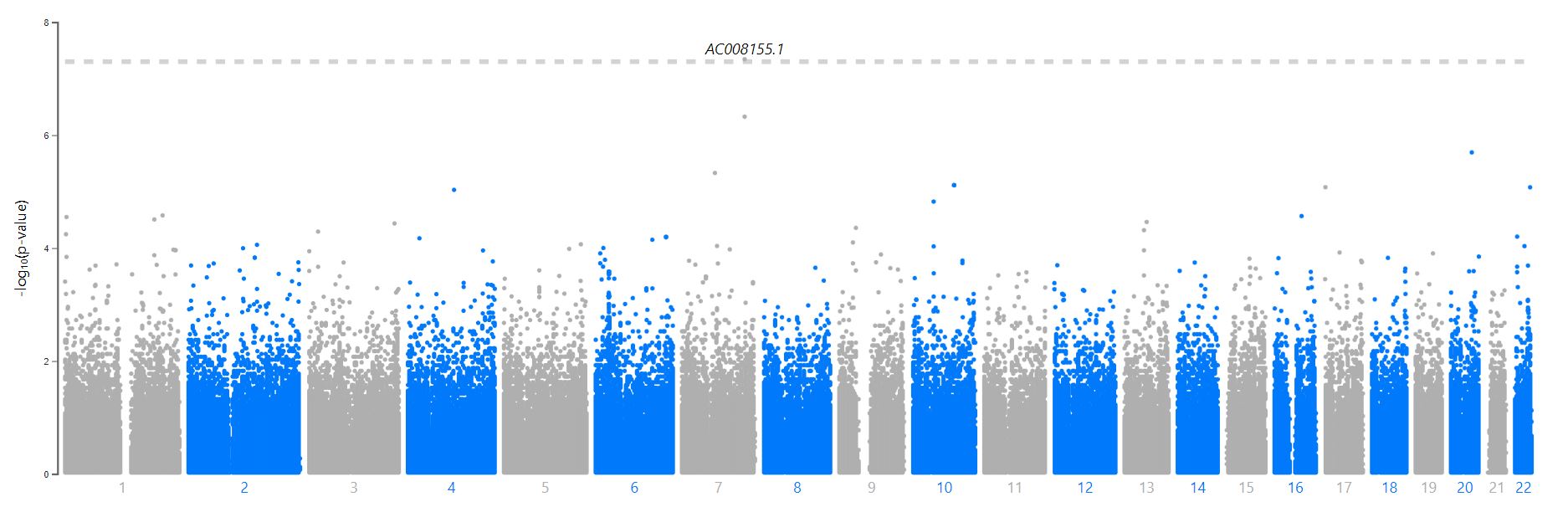


**Supplementary Figure 57. Upper Lip Circularity and GWAS association results.** The Manhattan plot shows the SNP associations to the measurement represented by the (-log_10_[P]) on the (y-axis) and the genotyped SNPs (each dot represents a SNP according to the chromosomal position (numbered from 1-22) on the (x-axis). The horizontal dashed grey line shows the genome-wide significance threshold which is (*p* = 5.0×10^−8^).


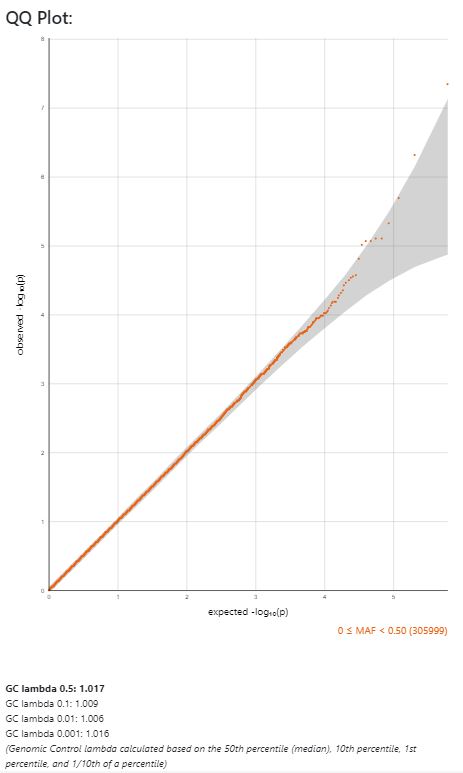


**Supplementary Figure 58. Q-Q Plot for the GWAS of Upper Lip Circularity measurement.** GC lambda 0.5: 1.017.


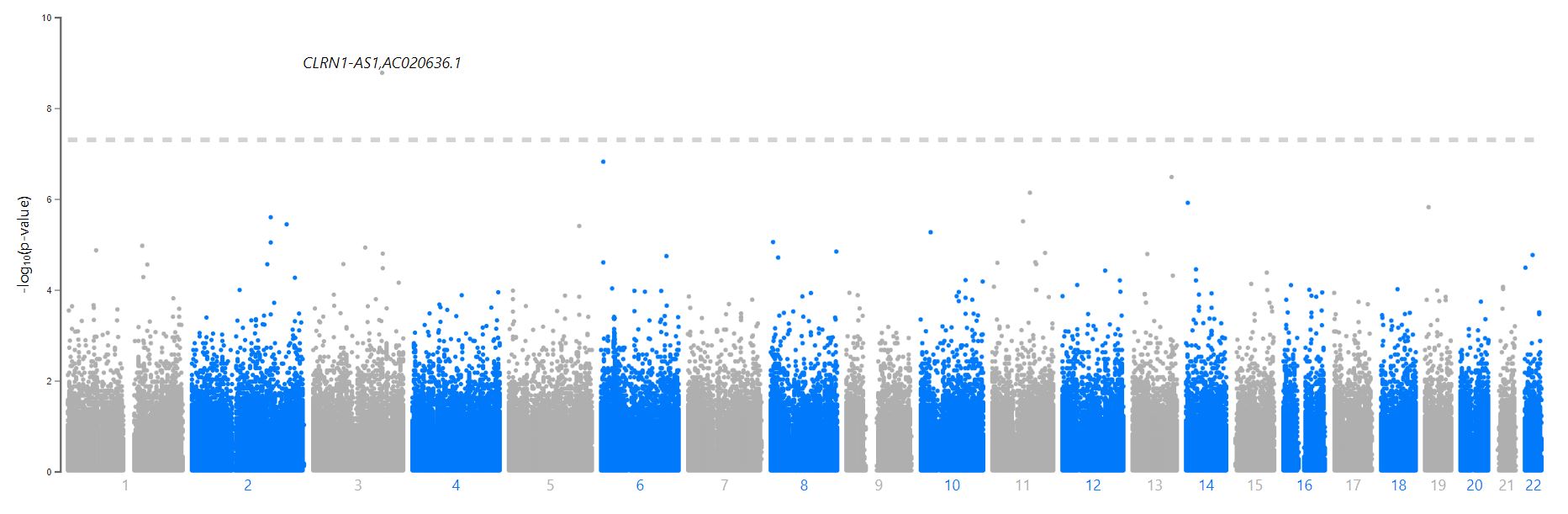


**Supplementary Figure 59. Outer Canthal, Nasal Angle and GWAS association results.** The Manhattan plot shows the SNP associations to the measurement represented by the (-log_10_[P]) on the (y-axis) and the genotyped SNPs (each dot represents a SNP according to the chromosomal position (numbered from 1-22) on the (x-axis). The horizontal dashed grey line shows the genome-wide significance threshold which is (*p* = 5.0×10^−8^).


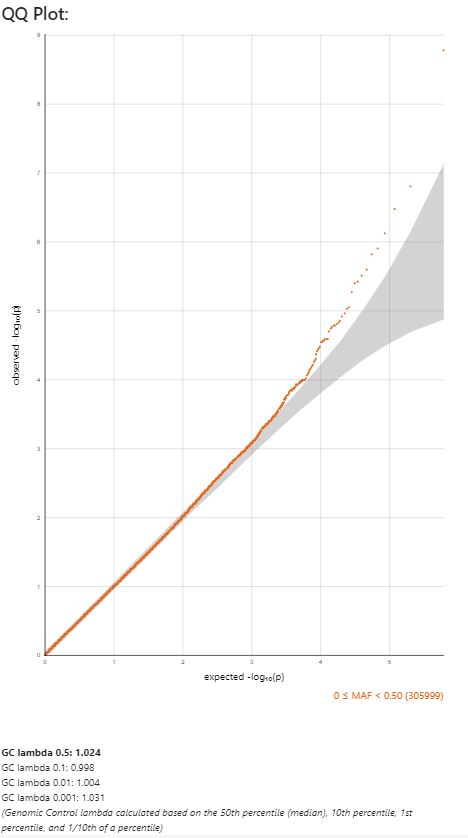


**Supplementary Figure 60. Q-Q Plot for the GWAS of Outer Canthal, Nasal Angle measurement.** GC lambda 0.5: 1.024.


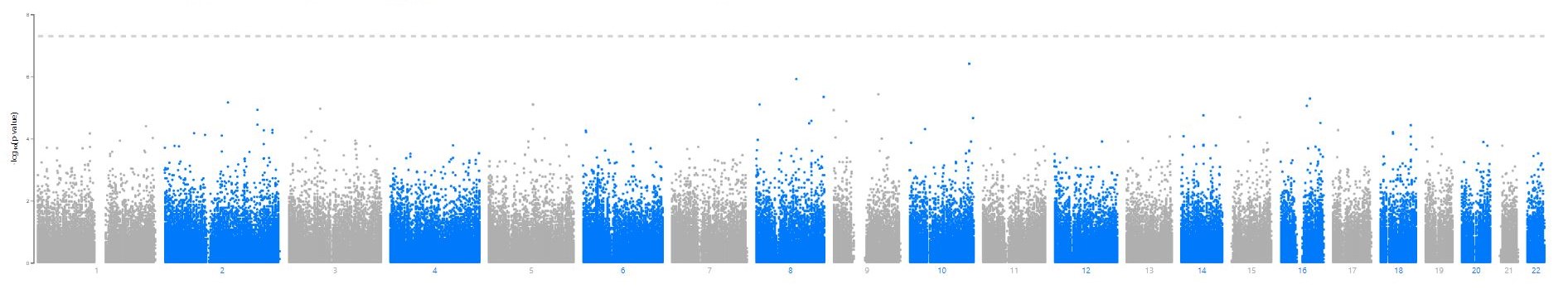


**Supplementary Figure 61. Cutaneous Lower Lip Height and GWAS association results.** The Manhattan plot shows the SNP associations to the measurement represented by the (-log_10_[P]) on the (y-axis) and the genotyped SNPs (each dot represents a SNP according to the chromosomal position (numbered from 1-22) on the (x-axis). The horizontal dashed grey line shows the genome-wide significance threshold which is (*p* = 5.0×10^−8^).


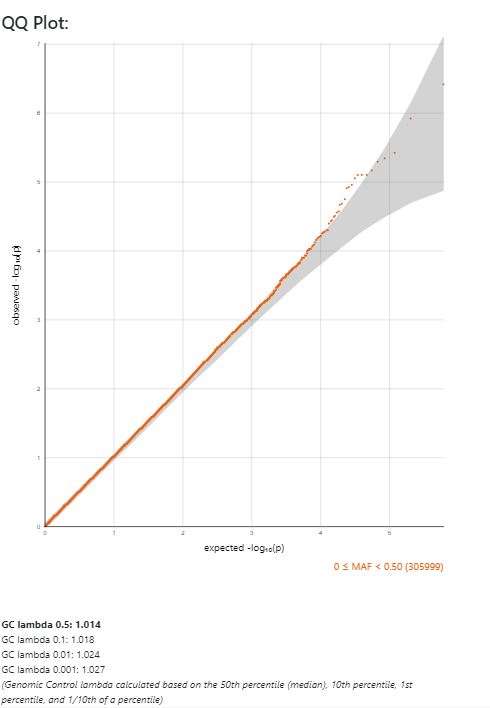


**Supplementary Figure 62. Q-Q Plot for the GWAS of Cutaneous Lower Lip Height measurement.** GC lambda 0.5: 1.014.


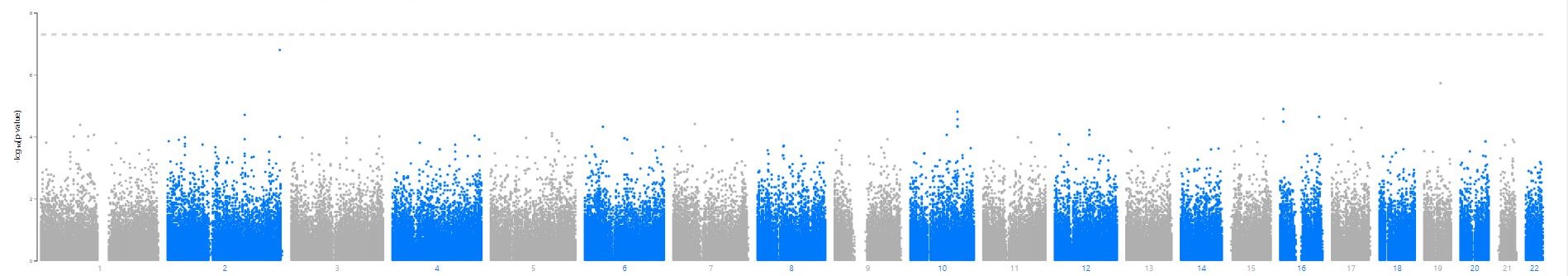


**Supplementary Figure 63. Left Nasal Ala Length and GWAS association results.** The Manhattan plot shows the SNP associations to the measurement represented by the (-log_10_[P]) on the (y-axis) and the genotyped SNPs (each dot represents a SNP according to the chromosomal position (numbered from 1-22) on the (x-axis). The horizontal dashed grey line shows the genome-wide significance threshold which is (*p* = 5.0×10^−8^).


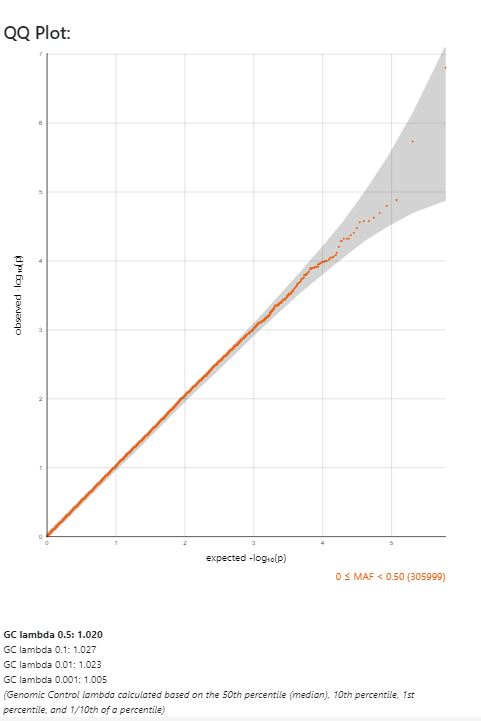


**Supplementary Figure 64. Q-Q Plot for the GWAS of Left Nasal Ala Length measurement.** GC lambda 0.5: 1.020.


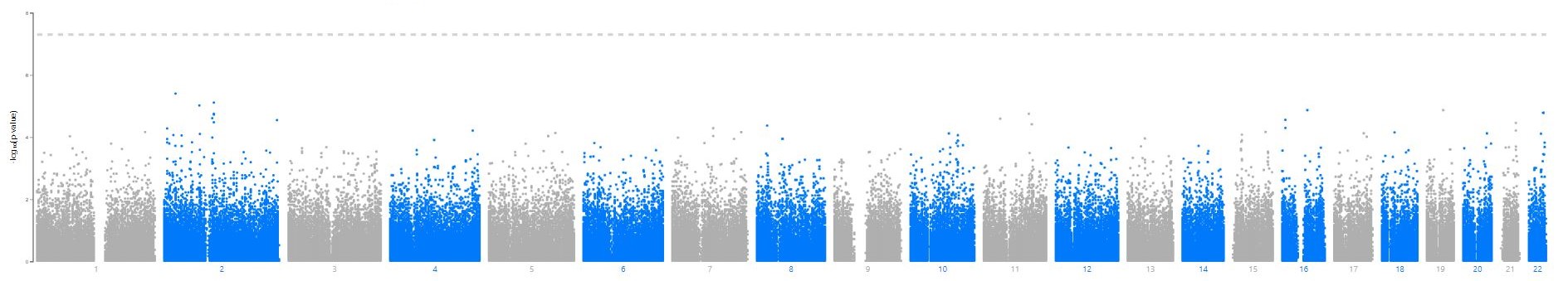


**Supplementary Figure 65. Right Nasal Ala Length and GWAS association results.** The Manhattan plot shows the SNP associations to the measurement represented by the (-log_10_[P]) on the (y-axis) and the genotyped SNPs (each dot represents a SNP according to the chromosomal position (numbered from 1-22) on the (x-axis). The horizontal dashed grey line shows the genome-wide significance threshold which is (*p* = 5.0×10^−8^).


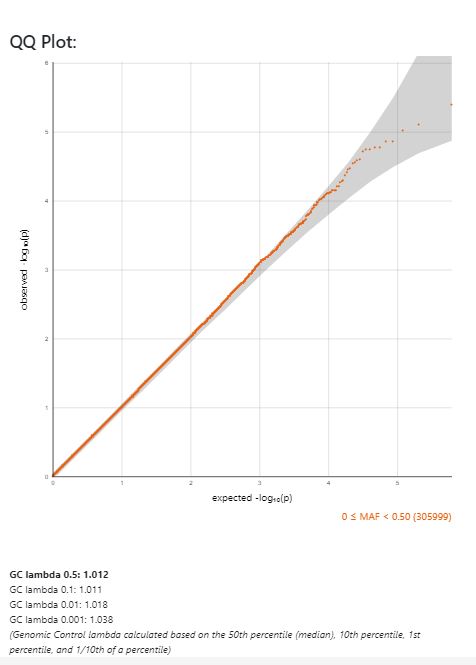


**Supplementary Figure 66. Q-Q Plot for the GWAS of Right Nasal Ala Length measurement.** GC lambda 0.5: 1.012.


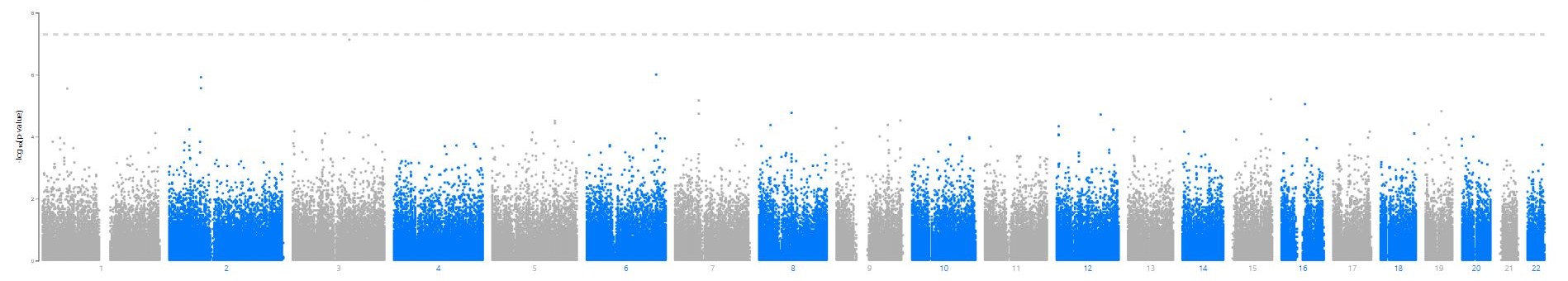


**Supplementary Figure 67. Nasofrontal Angle and GWAS association results.** The Manhattan plot shows the SNP associations to the measurement represented by the (-log_10_[P]) on the (y-axis) and the genotyped SNPs (each dot represents a SNP according to the chromosomal position (numbered from 1-22) on the (x-axis). The horizontal dashed grey line shows the genome-wide significance threshold which is (*p* = 5.0×10^−8^).


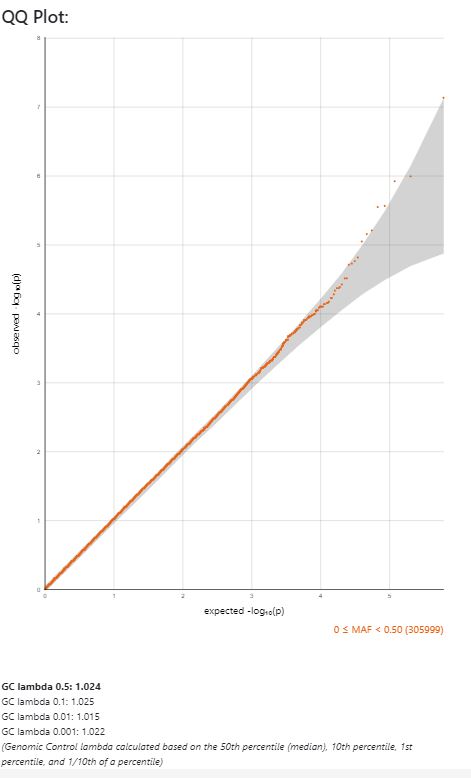


**Supplementary Figure 68. Q-Q Plot for the GWAS of Nasofrontal Angle measurement.** GC lambda 0.5: 1.024.


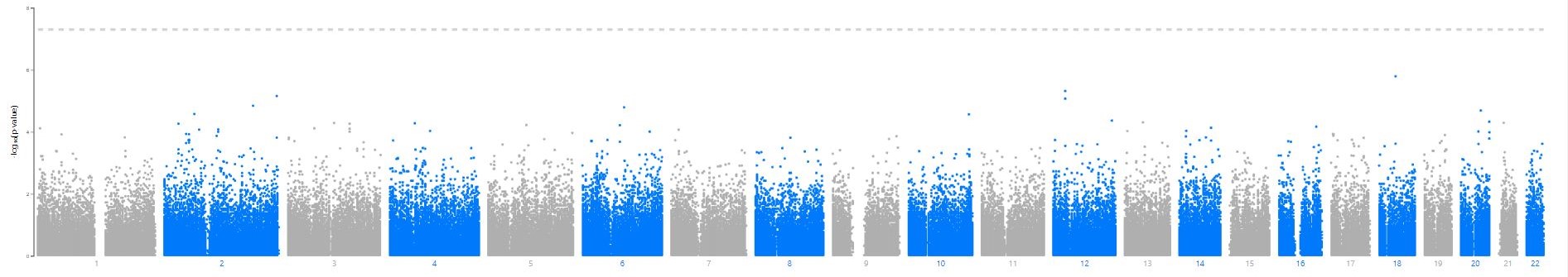


**Supplementary Figure 69. Nasal Angle and GWAS association results.** The Manhattan plot shows the SNP associations to the measurement represented by the (-log_10_[P]) on the (y-axis) and the genotyped SNPs (each dot represents a SNP according to the chromosomal position (numbered from 1-22) on the (x-axis). The horizontal dashed grey line shows the genome-wide significance threshold which is (*p* = 5.0×10^−8^).


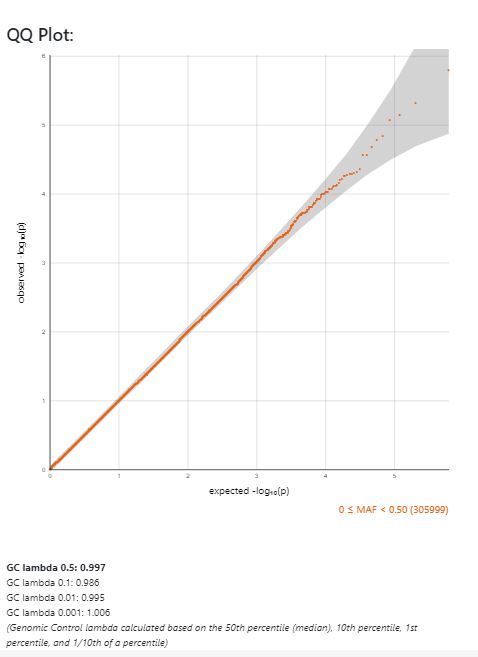


**Supplementary Figure 70. Q-Q Plot for the GWAS of Nasal Angle measurement.** GC lambda 0.5: 0.997.


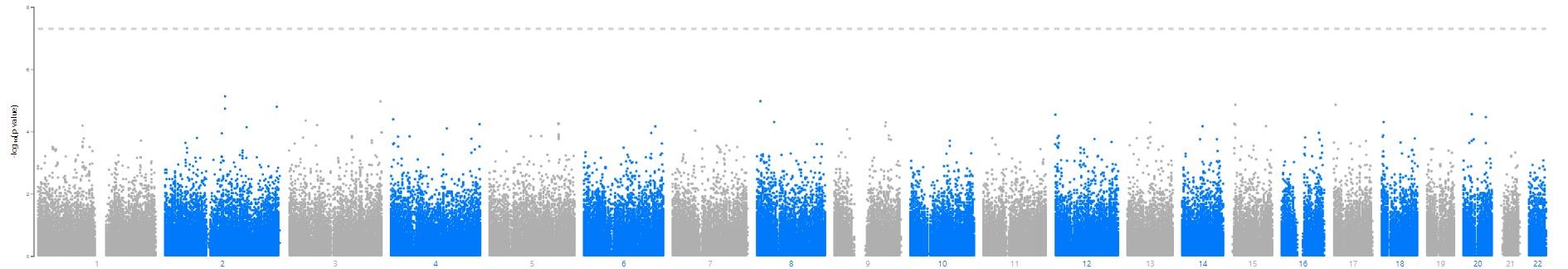


**Supplementary Figure 71. Nasolabial Angle A and GWAS association results.** The Manhattan plot shows the SNP associations to the measurement represented by the (-log_10_[P]) on the (y-axis) and the genotyped SNPs (each dot represents a SNP according to the chromosomal position (numbered from 1-22) on the (x-axis). The horizontal dashed grey line shows the genome-wide significance threshold which is (*p* = 5.0×10^−8^).


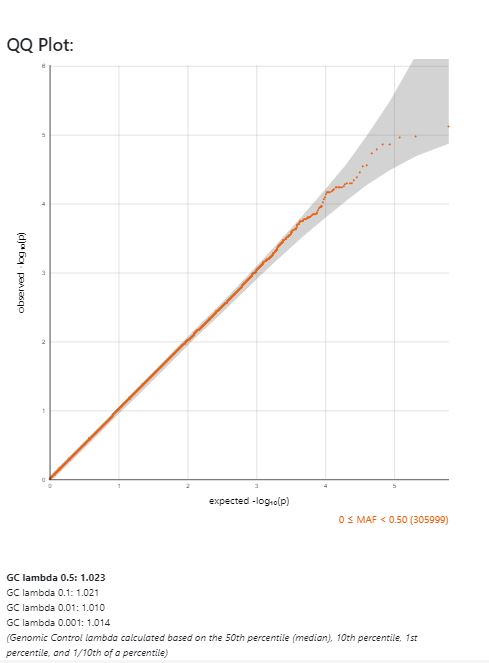


**Supplementary Figure 72. Q-Q Plot for the GWAS of Nasolabial Angle A measurement.** GC lambda 0.5: 1.023.


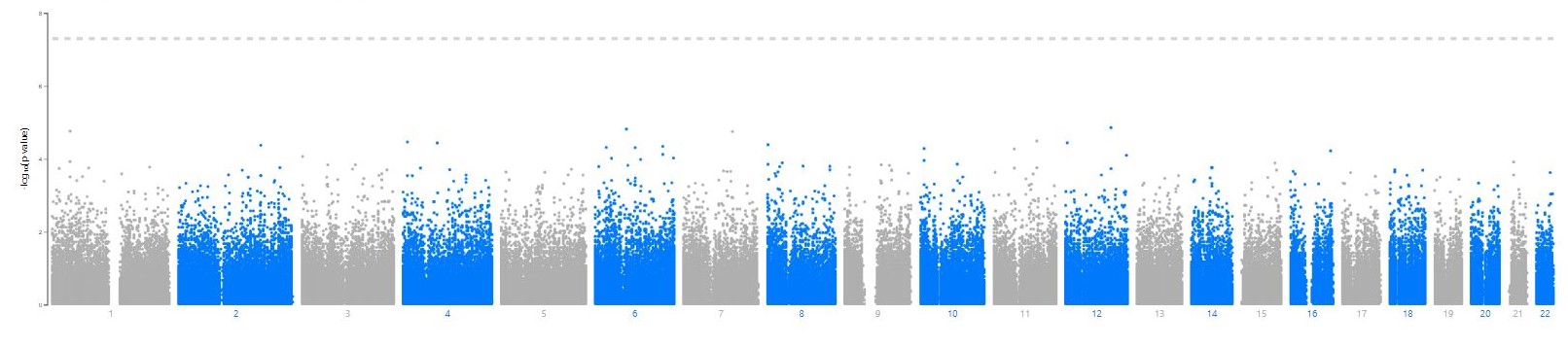


**Supplementary Figure 73. Total Facial Convexity and GWAS association results.** The Manhattan plot shows the SNP associations to the measurement represented by the (-log_10_[P]) on the (y-axis) and the genotyped SNPs (each dot represents a SNP according to the chromosomal position (numbered from 1-22) on the (x-axis). The horizontal dashed grey line shows the genome-wide significance threshold which is (*p* = 5.0×10^−8^).


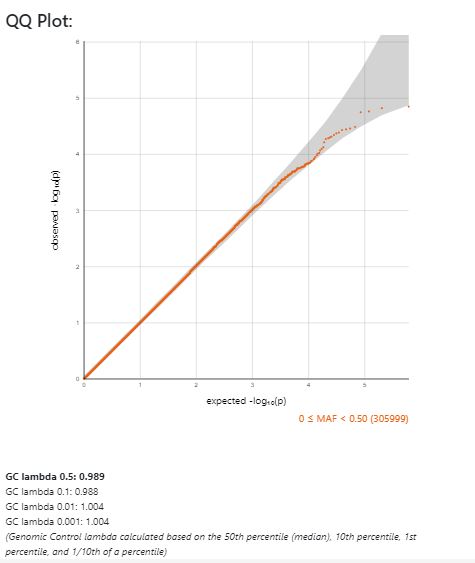


**Supplementary Figure 74. Q-Q Plot for the GWAS of Total Facial Convexity measurement.** GC lambda 0.5: 0.989.


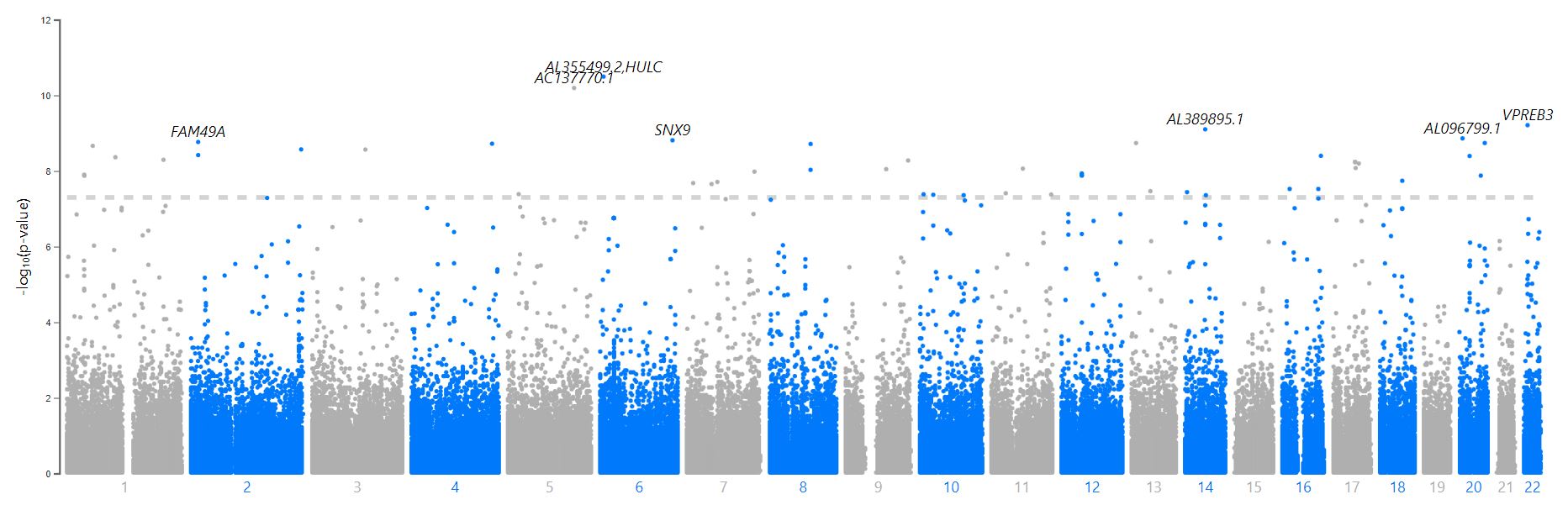


**Supplementary Figure 75. Nasolabial Angle D and GWAS association results.** The Manhattan plot shows the SNP associations to the measurement represented by the (-log_10_[P]) on the (y-axis) and the genotyped SNPs (each dot represents a SNP according to the chromosomal position (numbered from 1-22) on the (x-axis). The horizontal dashed grey line shows the genome-wide significance threshold which is (*p* = 5.0×10^−8^).


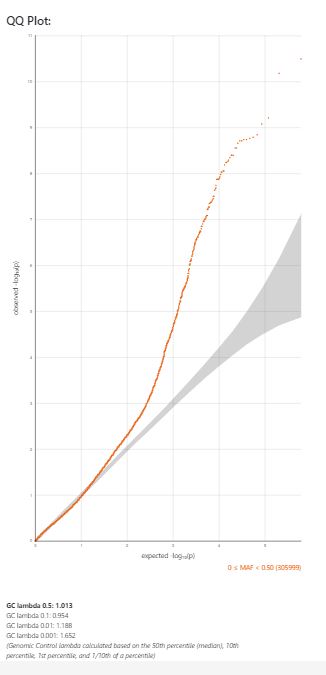


**Supplementary Figure 76. Q-Q Plot for the GWAS of Nasolabial Angle D measurement.** GC lambda 0.5: 1.013.


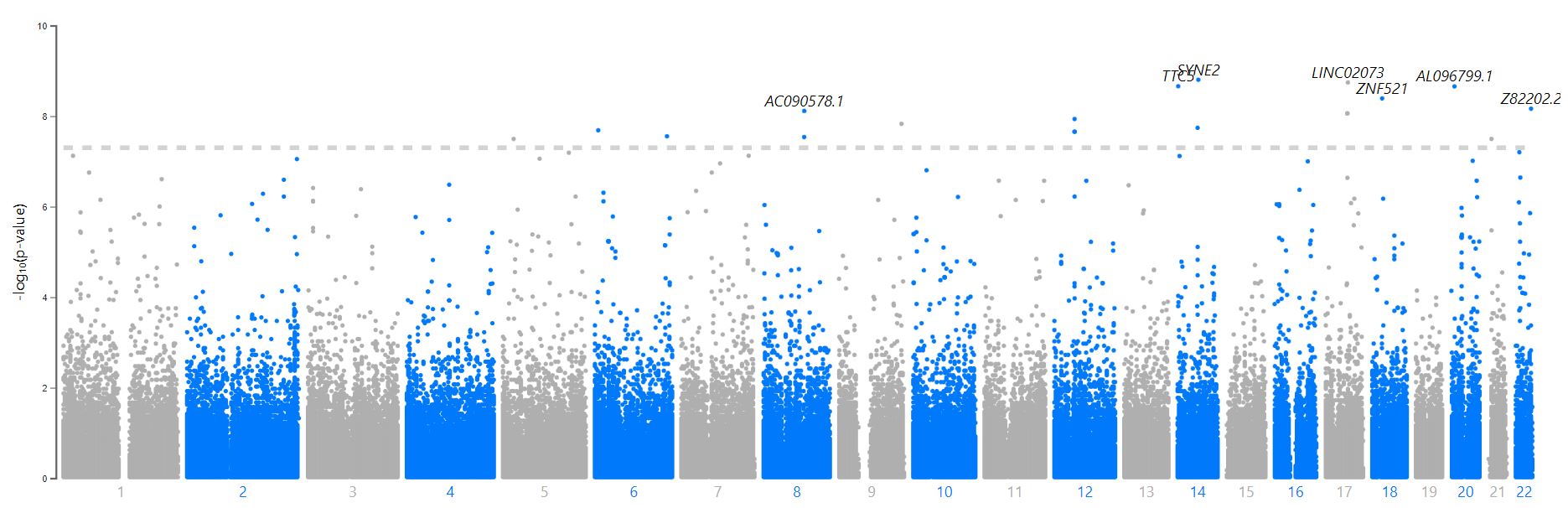


**Supplementary Figure 77. Inferior Facial Angle A and GWAS association results.** The Manhattan plot shows the SNP associations to the measurement represented by the (-log_10_[P]) on the (y-axis) and the genotyped SNPs (each dot represents a SNP according to the chromosomal position (numbered from 1-22) on the (x-axis). The horizontal dashed grey line shows the genome-wide significance threshold which is (*p* = 5.0×10^−8^).


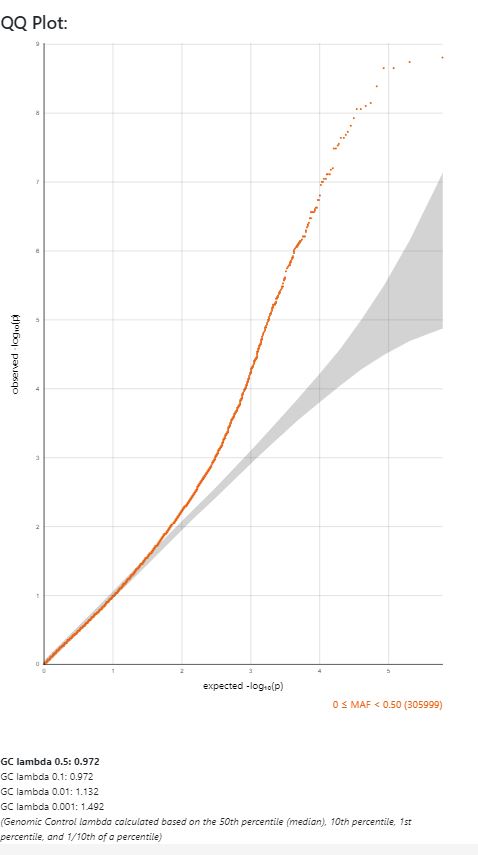


**Supplementary Figure 78. Q-Q Plot for the GWAS of Inferior Facial Angle A measurement.** GC lambda 0.5: 0.972.


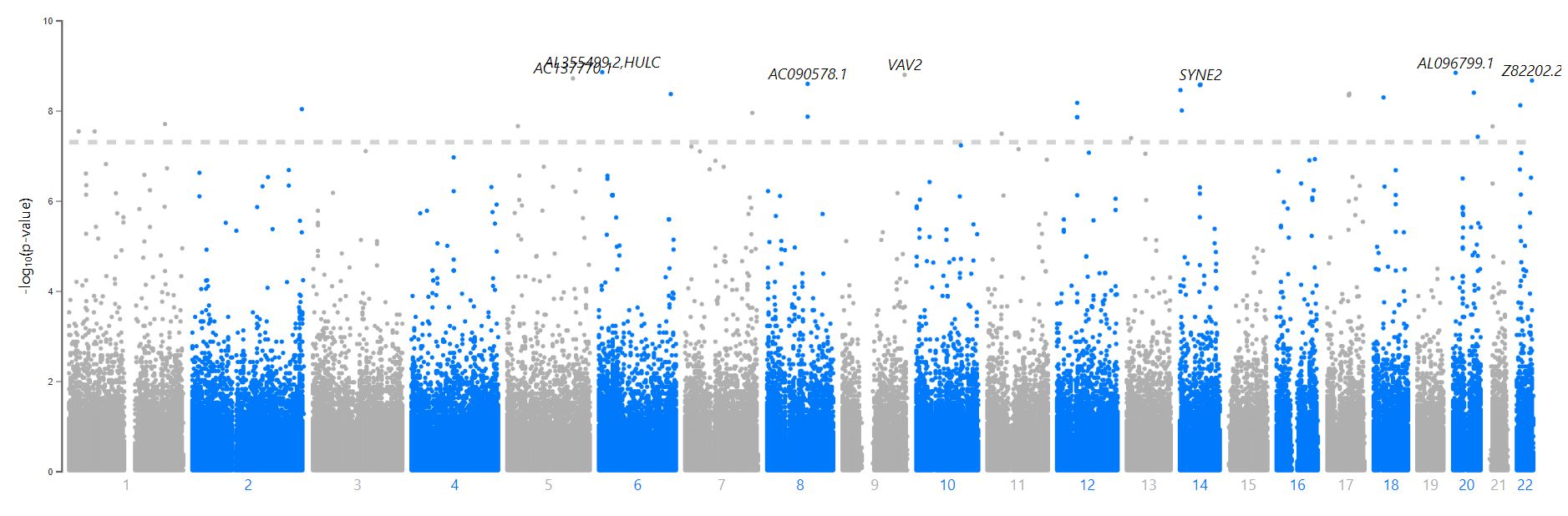


**Supplementary Figure 79. Inferior Facial Angle B and GWAS association results.** The Manhattan plot shows the SNP associations to the measurement represented by the (-log_10_[P]) on the (y-axis) and the genotyped SNPs (each dot represents a SNP according to the chromosomal position (numbered from 1-22) on the (x-axis). The horizontal dashed grey line shows the genome-wide significance threshold which is (*p* = 5.0×10^−8^).


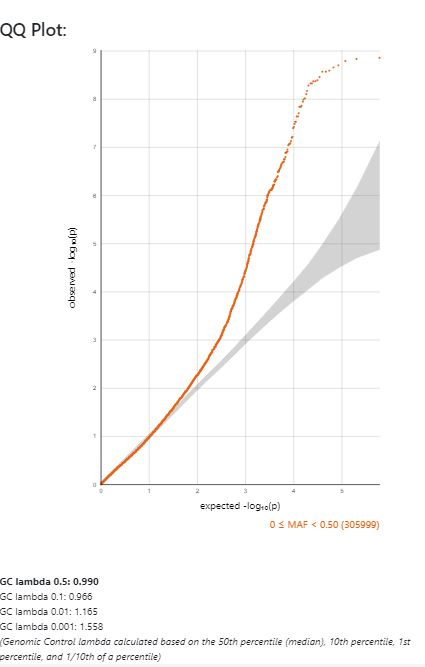


**Supplementary Figure 80. Q-Q Plot for the GWAS of Inferior Facial Angle B measurement.** GC lambda 0.5: 0.990.


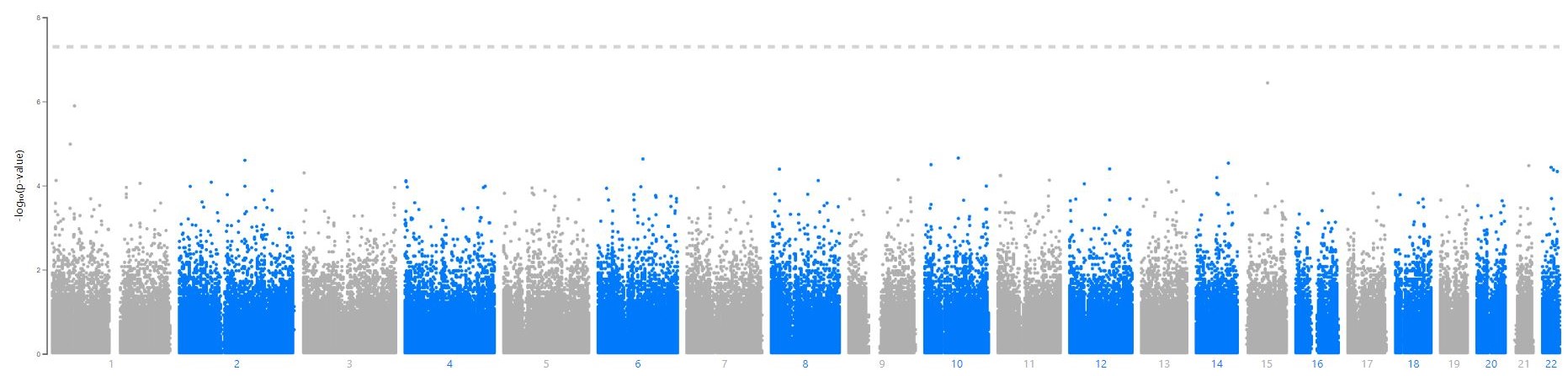


**Supplementary Figure 81. Inferior Facial Angle C and GWAS association results.** The Manhattan plot shows the SNP associations to the measurement represented by the (-log_10_[P]) on the (y-axis) and the genotyped SNPs (each dot represents a SNP according to the chromosomal position (numbered from 1-22) on the (x-axis). The horizontal dashed grey line shows the genome-wide significance threshold which is (*p* = 5.0×10^−8^).


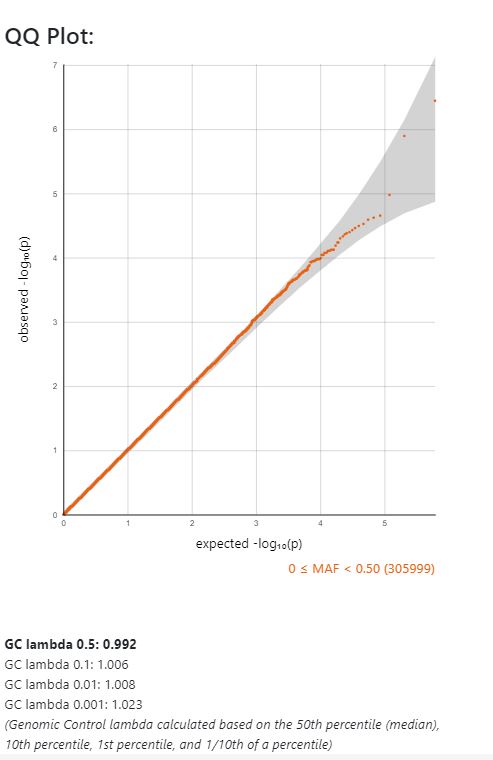


**Supplementary Figure 82. Q-Q Plot for the GWAS of Inferior Facial Angle C measurement.** GC lambda 0.5: 0.992.


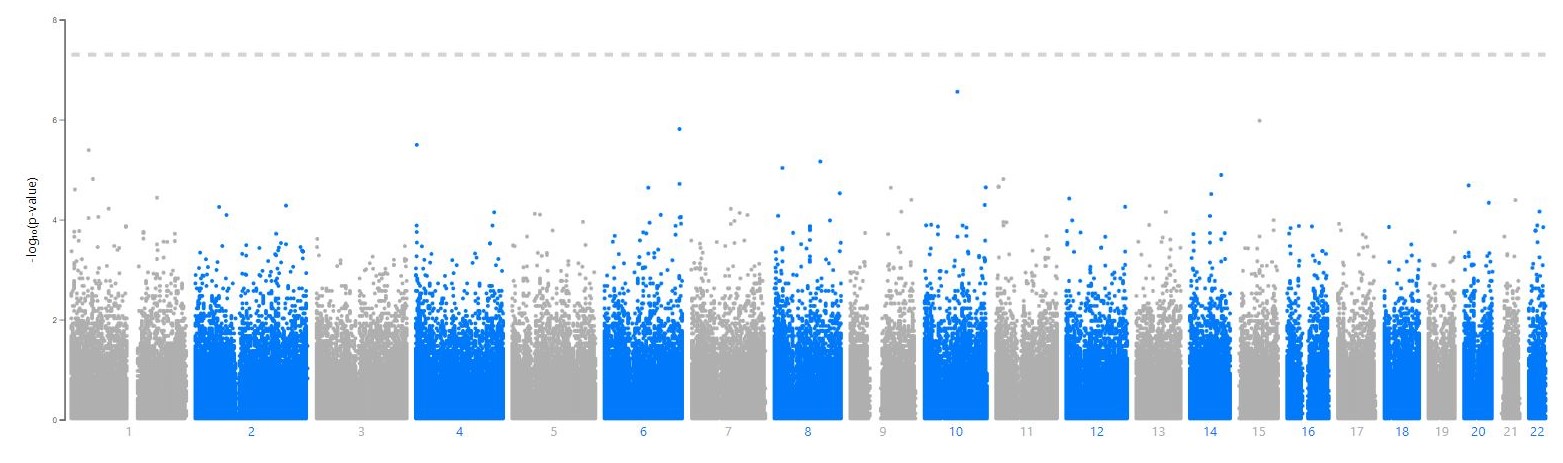


**Supplementary Figure 83. Inferior Facial Angle D and GWAS association results.** The Manhattan plot shows the SNP associations to the measurement represented by the (-log_10_[P]) on the (y-axis) and the genotyped SNPs (each dot represents a SNP according to the chromosomal position (numbered from 1-22) on the (x-axis). The horizontal dashed grey line shows the genome-wide significance threshold which is (*p* = 5.0×10^−8^).


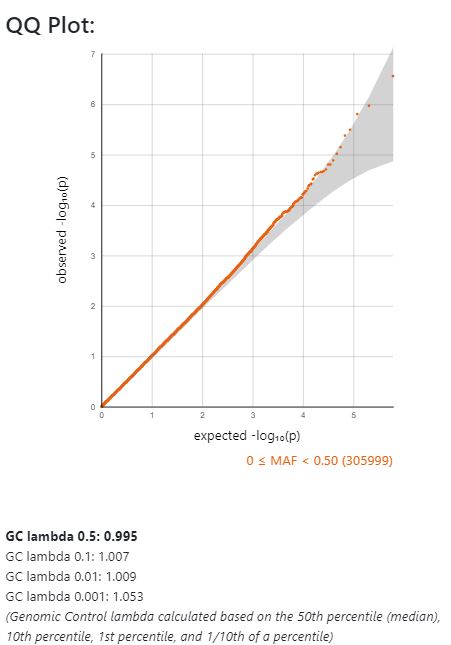


**Supplementary Figure 84. Q-Q Plot for the GWAS of Inferior Facial Angle D measurement.** GC lambda 0.5: 0.995.


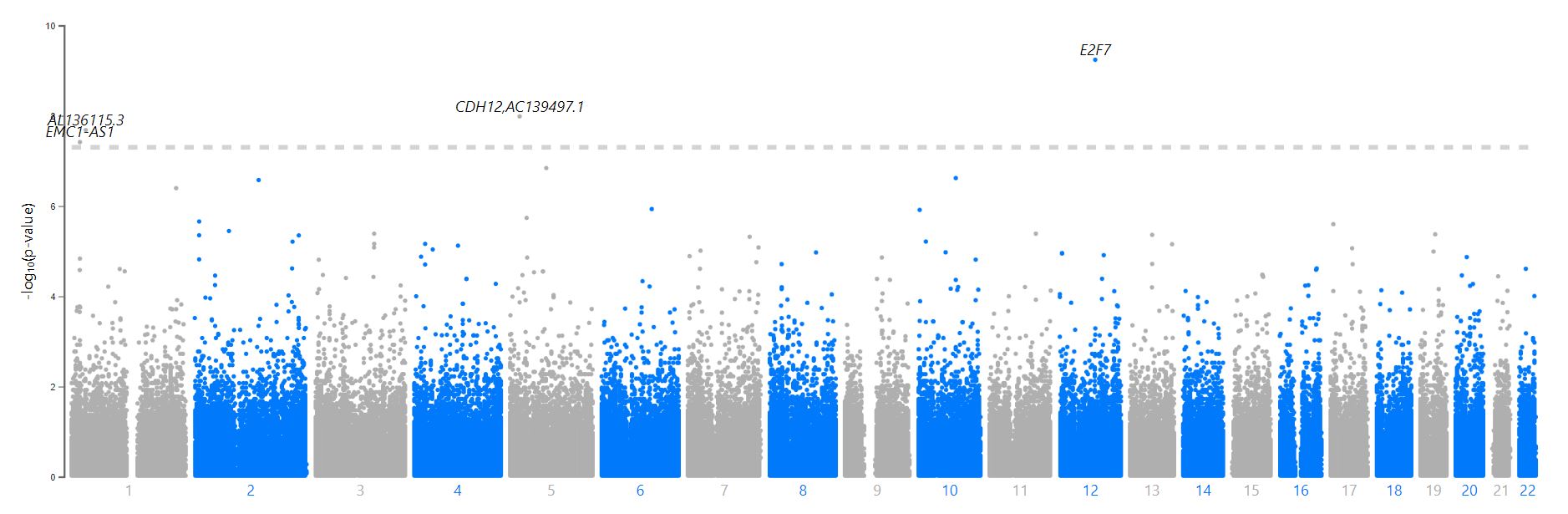


**Supplementary Figure 85. Left Orbital Protrusion and GWAS association results.** The Manhattan plot shows the SNP associations to the measurement represented by the (-log_10_[P]) on the (y-axis) and the genotyped SNPs (each dot represents a SNP according to the chromosomal position (numbered from 1-22) on the (x-axis). The horizontal dashed grey line shows the genome-wide significance threshold which is (*p* = 5.0×10^−8^).


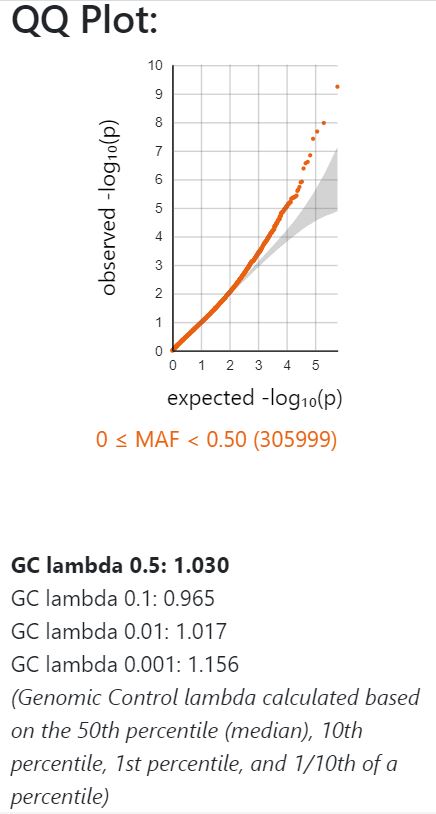


**Supplementary Figure 86. Q-Q Plot for the GWAS of Left Orbital Protrusion measurement.** GC lambda 0.5: 1.030.


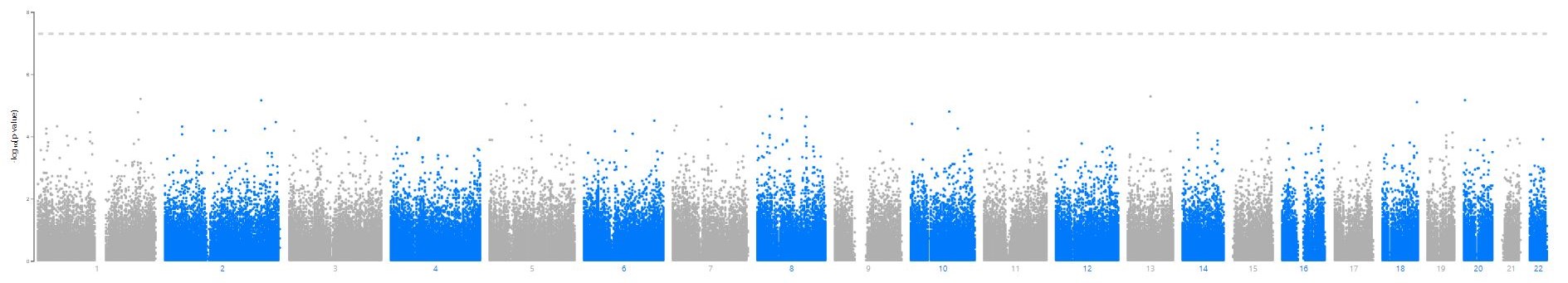


**Supplementary Figure 87. Right Orbital Protrusion and GWAS association results.** The Manhattan plot shows the SNP associations to the measurement represented by the (-log_10_[P]) on the (y-axis) and the genotyped SNPs (each dot represents a SNP according to the chromosomal position (numbered from 1-22) on the (x-axis). The horizontal dashed grey line shows the genome-wide significance threshold which is (*p* = 5.0×10^−8^).


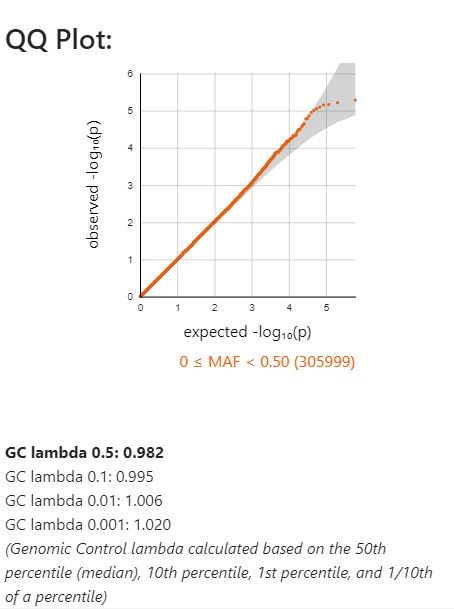


**Supplementary Figure 88. Q-Q Plot for the GWAS of Right Orbital Protrusion measurement.** GC lambda 0.5: 0.982.


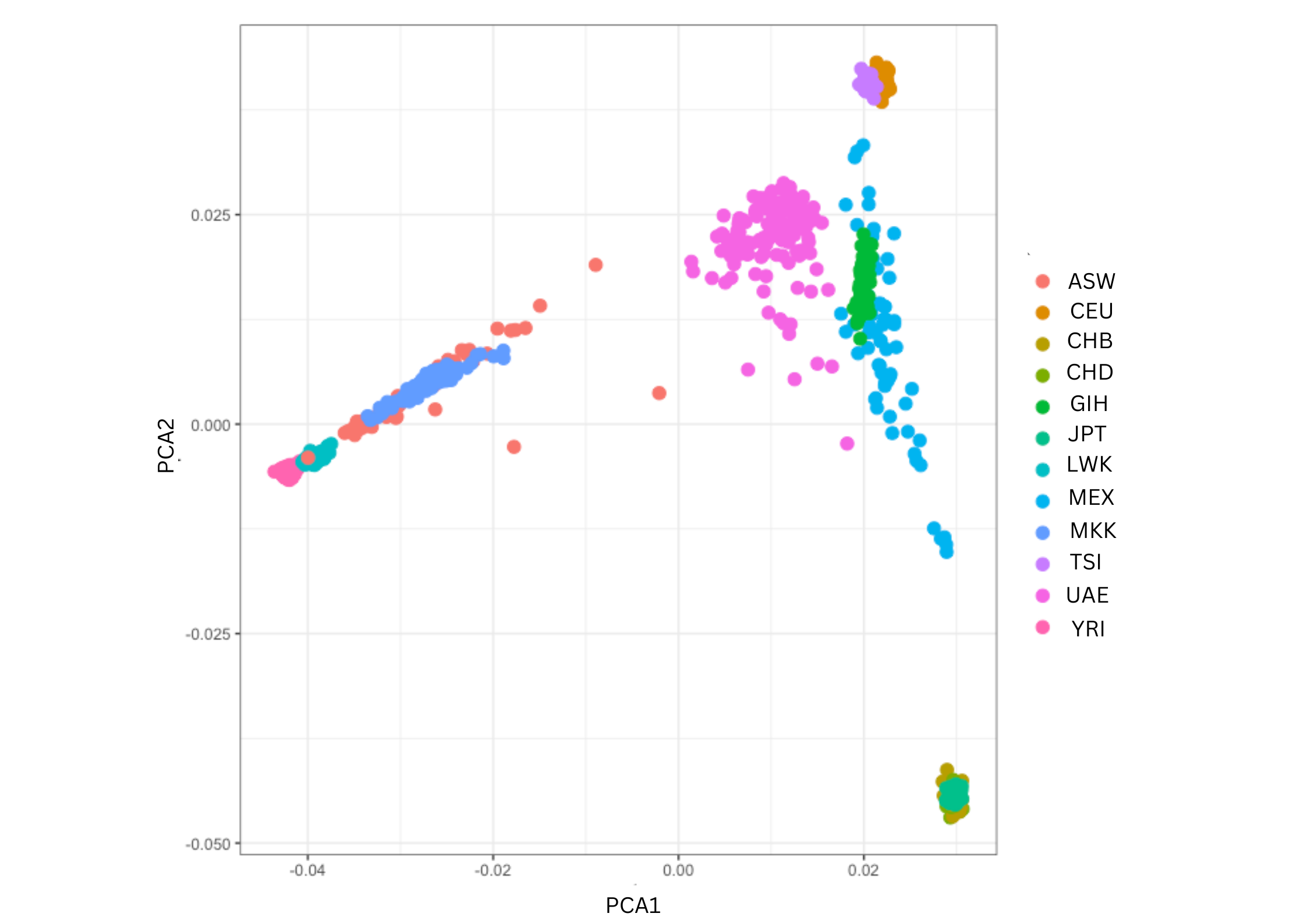


**Supplementary Figure 89. Admixture informed principal component analysis (PCA) plot of 159 participants from the UAE cohort and 1,115 participants from the HapMap 3 project.** This cohort (defined as UAE in the PCA plot) is represented as Fuschia, demonstrating a closely clustered pool. The population samples that were included in the PCA plot are: ASW (African ancestry in Southwest USA), CEU (Utah residents with Northern and Western European ancestry with CEPH collection), CHB (Han Chinese in Beijing, China), CHD (Chinese in Metropolitan Denver, Colorado), GIH (Gujarati Indians in Houston, TX), JPT (Japanese in Tokyo, Japan), LWK (Luhya in Webeye, Kenya), MEX (Mexican Ancestry in Los Angeles, CA)s, MKK (Maasai in Kinyawa, Kenya), TSI (Tuscany in Italia), UAE (United Arab Emirates cohort), YRI (Yoruba in Ibadan, Nigeria).
